# Thermogels Based on Block versus Gradient Terpolymers: Differences in the Nano- and Macro-Scale

**DOI:** 10.1021/acs.macromol.5c00821

**Published:** 2025-08-26

**Authors:** Anna P. Constantinou, Feifei Zheng, Lezhi Wang, Wenqi Xu, Qian Li, Beini Zhan, Joana Salvado Correia, Birsen SomuncuoĞlu, Stefano Da Vela, Christine M. Papadakis, Theoni K. Georgiou

**Affiliations:** † Department of Materials, 4615 Imperial College London, London SW7 2AZ, United Kingdom; ‡ Soft Matter Physics Group, Physics Department, TUM School of Natural Sciences, 84665 Technical University of Munich, James-Franck-Str. 1, 85748 Garching, Germany; § 128803 European Molecular Biology Laboratory, Hamburg Site c/o Deutsches Elektronen-Synchrotron, 22607 Hamburg, Germany

## Abstract

In the present study, the effect of the distribution of repeated units in copolymers based on oligo­(ethylene glycol) methyl ether methacrylate (A), *n*-butyl methacrylate (B), and di­(ethylene glycol) methyl ether methacrylate (C) is reported and discussed for the first time. Several linear structures have been synthesized via group transfer polymerization, varying from statistical to gradient to block structures. Notably, among the other linear architectures, we report the one-pot synthesis of a forced gradient terpolymer and the investigation of its thermoresponsive and gelation properties. It is proven that the distribution of repeated units along the polymer chain governs the solubility and gelation of the copolymers, with the ABC and gradient structures being the best-performing. The self-assembled structures and aggregation mechanisms differ strongly for the ABC triblock and the gradient terpolymer, as revealed by synchrotron small-angle X-ray scattering. The promising results of the gradient structure open a new era for designing novel copolymers with potential applications in the biomedical sector.

## Graphical abstract

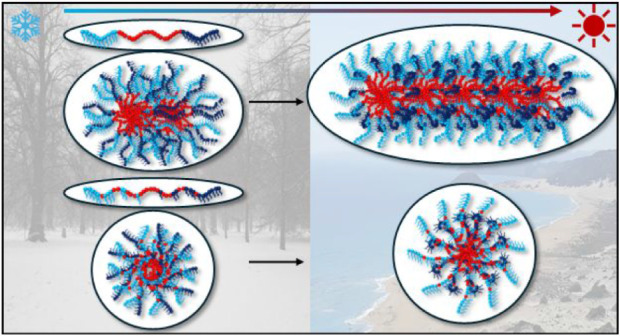

## Introduction

Stimuli-responsive polymers, also called “smart” polymers, are a fascinating class of polymers, as they possess tunable properties, the changes of which are triggered by changes in external stimuli. Extensively studied stimuli for biological applications include temperature and pH, and the polymers that respond to these stimuli are known as temperature-/thermoresponsive and pH-responsive, respectively. Of major interest is the category of thermoresponsive polymers because of its numerous applications in several sectors, such as the biomedical and manufacturing fields.

Thermoresponsive polymers are subdivided into polymers with (i) upper critical solution temperature (UCST) and (ii) lower critical solution temperature (LCST). These are polymers whose solubility increases and decreases as a function of temperature, respectively. Under the appropriate internal and external conditions, i.e., polymer structure and environment, thermoresponsive polymers can form a 3D network, widely known as a thermoresponsive gel or thermogel. A well-known UCST polymer in everyday life is gelatin,^1^ whose use in the food industry has contributed to its popularity. In the biomedical sector, LCST polymers are preferable because they are soluble in a solvent at low temperatures, e.g., room temperature, while they can form a gel at higher temperatures, e.g., body temperature. Owing to this property, drugs and/or cells can be dissolved in the polymer solution at room temperature, while the mixture will exist in its gel state at body temperature. These concepts are applied in the tissue engineering and drug delivery fields, when the incorporation of cells and drugs is concerned, respectively.^2^ The use of thermoresponsive gels offers the advantage of new tissue regeneration without the need for invasive surgery as well as topical and controlled release.^3^ Some thermoresponsive gels are also being used as 3D printable materials, because of their shear-thinning properties,^4,5^ and they are used either to enable the printing of nonprintable materials or for bioprinting structures for tissue engineering. In either application, the solution phase facilitates easy loading into a syringe. Therefore, thermoresponsive gels with LCST behavior have attracted significant scientific interest over the past few decades.

Polymer chemists have played a major role in this field. Using “living” and controlled polymerization techniques as their tools, they have fabricated novel synthetic thermoresponsive polymers. Thus, limitless possibilities have been unlocked, and novel combinations of repeated units embedded in complex architectures are now feasible.^6^ The most well-studied thermoresponsive units are *N*-isopropylacrylamide (NIPAAm),^7^ ethylene glycol (EG) and its derivatives,^8,9^ 2-(dimethylamino)­ethyl methacrylate (DMAEMA),^8,10,11^ and oxazolines/oxazines.^12,13^ These units have been copolymerized with other units either randomly or in more complicated and well-defined structures. Examples include linear architectures with block arrangements of repeated units, such as di-,^14–16^ tri-,^17,18^ tetra-,^19–21^ penta-,^22–24^ hepta-,^24^ and nonablock copolymers,^24^ as well as hyperbranched and graft architectures.^26^ The synthesis has been facilitated through “living” and controlled polymerization techniques, such as reversible addition–fragmentation chain-transfer (RAFT) polymerization, group transfer polymerization (GTP), atom transfer radical polymerization (ATRP), and living cationic ring-opening polymerization (CROP).

Our group carried out several studies on the synthesis of thermoresponsive polymers and the investigation of their self-assembly and gelation properties. All of the copolymers were synthesized via GTP, as it allows the synthesis of well-defined multiple block copolymers on the same day. Most of the studies investigated amine-containing thermoresponsive polymers, either DMAEMA or 2-(diethylamino)­ethyl methacrylate (DEAEMA).^6^ Focusing on the effect of the distribution of repeated units, we investigated diblock, triblock, and tetrablock terpolymers, where A is based on a hydrophilic EG-based methacrylate unit, such as oligo­(ethylene glycol) methyl ether methacrylate with an average *M*_n_ of 300 g mol^–1^ (OEGMA300), B is based on the hydrophobic *n*-butyl methacrylate (BuMA), and C on either DMAEMA or DEAEMA. Recently, we have published on ABC triblock terpolymers of varying compositions based on a novel combination of repeated units, in which A and B were OEGMA300 and BuMA, respectively, while the thermoresponsive unit is di­(ethylene glycol) methyl ether methacrylate (DEGMA).^27,28^

An interesting linear polymer structure that has attracted attention due to its potential desirable properties is the gradient structure. In addition, when gradient copolymers are concerned, one-step synthesis may be achieved owing to the different reactivity ratios of the monomers.^29^ Thermoresponsive gradient copolymers have been synthesized and investigated by several groups, and they are largely associated with the names of Matyjaszewski,^30–32^ Hoogenboom,^29,33–40^ Schubert,^39–43^ Harrisson,^44–50^ and their coworkers. In these studies, thermoresponsive gradient copolymers based on oxazolines/oxazines, (meth-)­acrylate, acrylamide, and styrene-based units have been synthesized via CROP, ATRP, or RAFT polymerization and investigated in terms of their properties. In an interesting study by Kaberov et al., fluorinated gradient and block poly­(oxazoline) polymers were investigated for their applicability as ^19^F MRI contrast agents.^51^ The gradient counterparts were the best-performing in ^19^F MRI when compared to the block copolymers, as they showed the best ^19^F MRI signal-to-noise ratio.^51^ In addition to the several publications on gradient copolymers based on two types of repeated units, Lambermont-Thijs et al. reported the unexpected synthesis of a gradient terpolymer, i.e., a polymer with three repeated units which are distributed in a gradient rate along the polymer chain, based on 2-methyl-2-oxazoline, 4-ethyl-2-butyl-2-oxazoline, and 2-phenyl-2-oxazoline via CROP.^52^

To the best of our knowledge, a thermoresponsive gradient terpolymer has yet to be investigated, and therefore, herein, we report its controlled synthesis and interesting thermoresponsive properties. Most importantly, the synthesis is performed via one-pot forced GTP, which is an industrially discovered and thus industrially applicable, time- and cost-effective polymerization technique.^53–55^ To compare this novel copolymer, several linear copolymers with varying distributions of repeated units were also fabricated for comparison, including statistical and block structures. Therefore, in this study, the effect of the distribution of repeated units on the thermoresponsive properties of polymers based on BuMA (B unit), OEGMA300 (A unit), and/or DEGMA (C unit) has been studied and presented. The molar mass (MM) was kept constant at 8300 g mol^–1^ to investigate their differences in self-assembly and gelling behavior. To explore the effect of the block structure in greater depth, in the absence of molar mass effects, several block copolymers were synthesized and investigated. More specifically, two triblock copolymers, namely, ABA and CBC, a triblock terpolymer with two statistical hydrophilic outer blocks and a hydrophobic central block, i.e., (AC)­B­(AC), an ABC and a BAC triblock terpolymer, and two tetrablock terpolymers with ACBC and BABC linear structures were synthesized. The polymers are categorized into two families, depending on their hydrophobic BuMA content: (a) 30 w/w% and (b) 35 w/w%, as depicted in Figure 1 below. In Figure 1, the units of OEGMA300, BuMA, and DEGMA are represented as light blue, red, and dark blue spheres, respectively.

**Figure 1:**
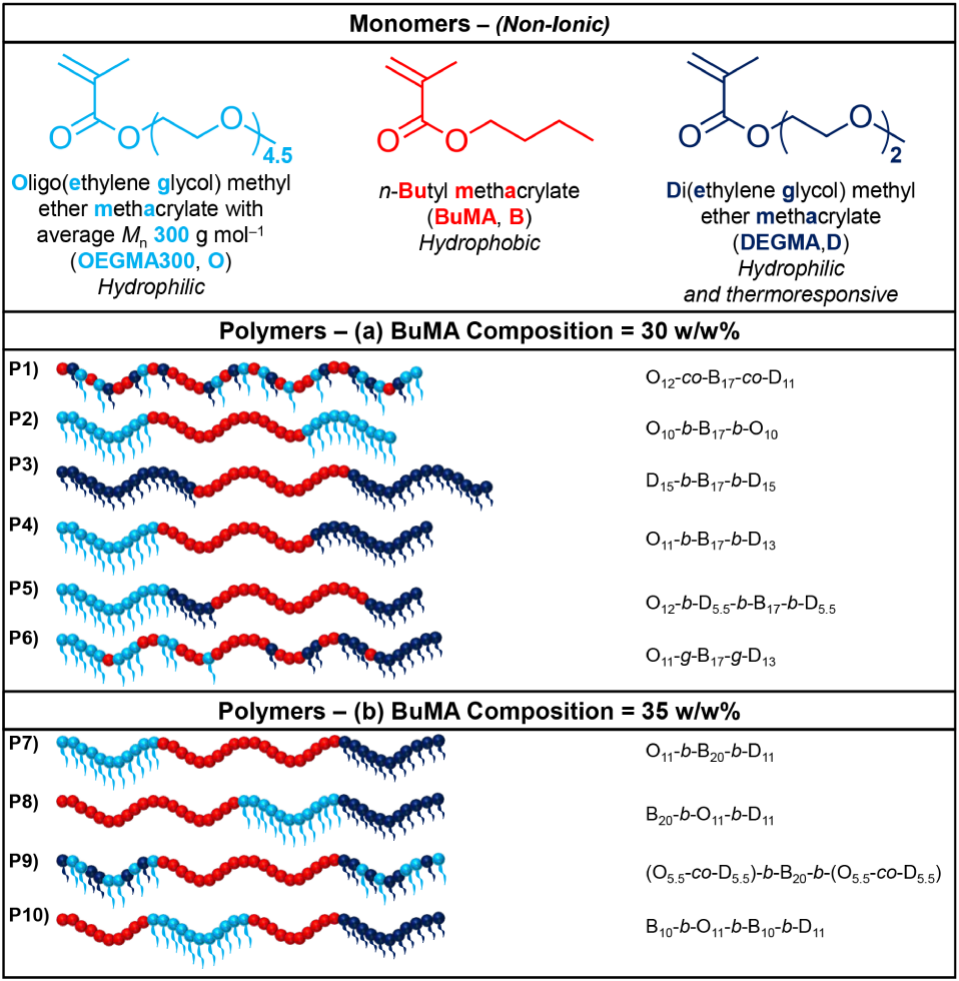
Chemical structures, names, and abbreviations of the three monomers (top) and schematic representation of the polymer structures studied (bottom); light blue, red, and dark blue spheres represent the hydrophilic OEGMA300, hydrophobic BuMA, and thermoresponsive DEGMA units, respectively.

## Experimental Section

### Materials

Sigma-Aldrich, UK, was the provider of BuMA (99%), DEGMA (95%), OEGMA300 (94%), 2,2-diphenyl-1-picrylhydrazyl hydrate (DPPH), basic aluminum oxide (Al_2_O_3_·KOH), calcium hydride (CaH_2_, ≥90%), deuterated chloroform (chloroform-*d*, 99.8 atom %D, CDCl_3_), methyltrimethylsilyl dimethylketene acetal (MTS, 95%), tetrahydrofuran (THF, inhibitor-free, HPLC grade, ≥99.9%, GTP solvent), and phosphate-buffered saline (PBS) tablets. Acros Organics, UK, was the supplier of benzoic acid and tetrabutyl ammonium hydroxide (40% in water). Fisher Scientific was the distributor of THF (gel permeation chromatography grade, BHT stabilized), PBS (10× solution), poly­(tetrafluoroethylene) (PTFE) hydrophilic syringe filters (0.45 μm pore size, 25 mm diameter), and nylon syringe filters (0.45 μm pore size, 25 mm in diameter). VWR Chemicals was the provider of *n*-hexane, while Fluka, Sigma-Aldrich, UK, was the provider of poly­(methyl methacrylate) (PMMA) standard samples (molar mass (MM) = 2000, 4000, 8000, 20000, 50000, 100000 Da).

### Preparation for Polymerization

Monomer purification was carried out as described below. BuMA and DEGMA monomers, which are of low MM, were purified by (i) passing through basic alumina, (ii) adding DPPH, (iii) adding CaH_2_, and (iv) vacuum distillation. These steps were carried out to (i) remove acidic impurities (e.g., methacrylic acid) and the stabilizer, (ii) prevent undesired polymerization prior to GTP, (iii) ensure a humidity-free environment, and (iv) ensure the purity of the compound by removing the DPPH, Ca­(OH)_2_, and unreacted CaH_2_. On the other hand, the OEGMA300 monomer, which is of higher MM and thus of high viscosity, was purified via a slightly different procedure. To reduce its viscosity and ensure ease in handling, the OEGMA300 monomer was mixed with THF (50 vol %), and it was then passed through basic alumina, followed by the addition of CaH_2_. No DPPH was added because OEGMA300 cannot be distilled. The produced Ca­(OH)_2_ and unreacted CaH_2_ were removed via direct filtration with PTFE filters in the polymerization flask. The GTP catalyst, tetrabutylammonium bibenzoate (TBABB), was synthesized and purified as previously reported,^56^ while the GTP initiator, MTS, was vacuum-distilled before polymerization. A solvent purification system equipped with an activated alumina column (PureSolv Micro 100 Liter, purchased from Sigma-Aldrich) was used to purify the THF solvent. The glassware used for distillation and polymerization was dried in an oven at 140 °C overnight.

### Group Transfer Polymerization

All of the copolymers were synthesized via GTP, either by sequential or simultaneous addition of the monomers, depending on the distribution of the repeated units within the linear polymer chain. More specifically, sequential addition was implemented for the synthesis of block copolymers in which each block consists only of one type of repeated unit; these include the ABA (Polymer 2), CBC (Polymer 3), ABC (Polymers 4 and 7), ACBC (Polymer 5), BAC (Polymer 8), and BABC (Polymer 10) linear structures. For the synthesis of the statistical terpolymer (Polymer 1), the monomers reacted simultaneously as they were injected prior to the addition of the initiator. In the case of the triblock terpolymer with two statistical outer blocks (Polymer 9), the OEGMA300 and DEGMA monomers were injected simultaneously to form the first statistical block, followed by the addition of BuMA, which led to the formation of the (AC)B diblock terpolymer, and then simultaneous injection of the OEGMA300 and DEGMA monomers to allow the formation of the final (AC)B (AC) triblock terpolymer. For all these copolymers, the quantities varied to achieve the desired composition and MM. The most complicated and yet interesting linear structure studied is the gradient terpolymer (Polymer 6), the synthesis of which is described below and schematically illustrated in Figure 2. It is noteworthy that the polymerization yield is quantitative; thus, intermediate purification steps are not required for sequential GTP. The final copolymers were purified via precipitation in cold *n*-hexane, and they were vacuum-dried for a week.

**Figure 2:**
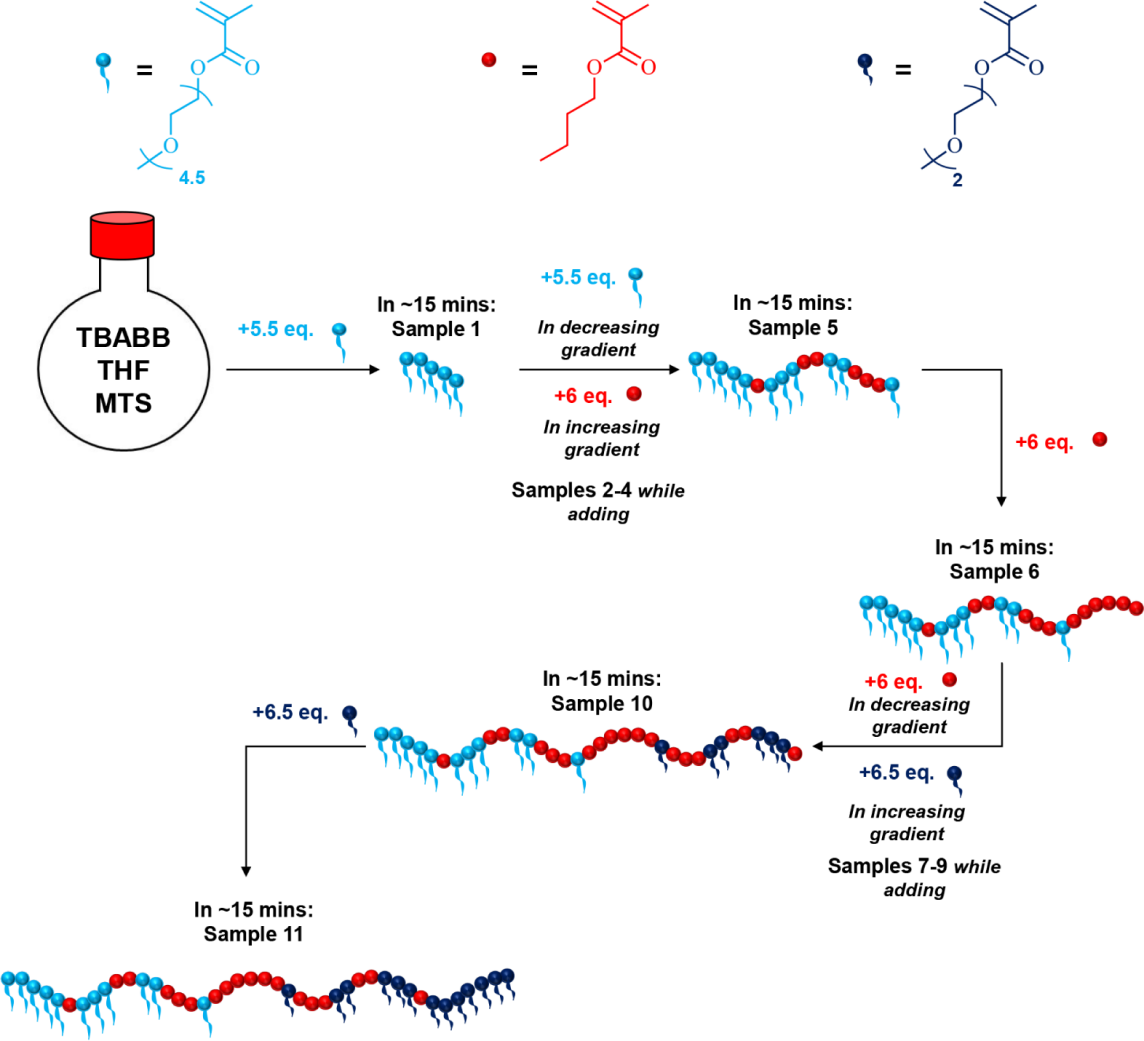
Schematic showing the synthetic procedure followed for the GTP of the gradient terpolymer (Polymer 6). TBABB, THF, and MTS are the catalyst, solvent, and initiator used during this GTP.

As shown in Figure 2, the synthesis of the gradient terpolymer was completed in five steps. First, an OEGMA300 solution (50 vol % in THF, 8.5 mL, 4.4 g, 15 mmol, 5.5 equiv) was injected into a 250 mL sealed round-bottom flask that contained the catalyst TBABB (∼10 mg), the solvent THF (60 mL), and the initiator MTS (0.55 mL, 0.47 g, 3 mmol, 1 equiv) under an inert argon atmosphere. The mixture was allowed to polymerize for around 15 min, and then, two aliquots of ∼0.1 mL each (Sample 1) were withdrawn for analysis using size exclusion chromatography (SEC) and proton nuclear magnetic resonance (^1^H NMR) spectroscopy. In the second step of the synthesis, 8.5 mL of an OEGMA300 solution (4.4 g, 15 mmol, 5.5 equiv) and 2.5 mL of a BuMA monomer (2.2 g, 16 mmol, 6 equiv) were simultaneously injected at descending and ascending rates, respectively. This addition was performed in three stages, after which samples were withdrawn for SEC and NMR analysis (Samples 2 to 4). After the complete consumption of the monomers (∼15 min later), SEC and NMR samples were obtained (Sample 5). As a third step, a BuMA monomer was added (2.5 mL, 2.2 g, 16 mmol, 6 equiv), and in ∼15 min, samples were withdrawn (Sample 6). For the fourth step, a second gradient block was synthesized by simultaneously injecting BuMA (2.5 mL, 2.2 g, 16 mmol, 6 equiv) and DEGMA (3.3 mL, 3.3 g, 18 mmol, 6.5 equiv) at descending and ascending rates, respectively. Similar to step 2, the addition was completed in three stages, during which samples were withdrawn (Samples 7 to 9). Once the polymerization was completed (in ∼15 min), two aliquots for SEC and NMR analysis were withdrawn (Sample 10). In the final synthetic step, a DEGMA monomer was injected into the polymerization flask (3.3 mL, 3.3 g, 18 mmol, 6.5 equiv), and after its complete consumption, a final sample was obtained (Sample 11). It should be noted that the monomer additions were performed manually, and a timer was used during the synthesis of the gradient terpolymer to control the speed of addition. The exothermic GTP was confirmed, and the temperature changes recorded are as follows: (i) from 22.5 to 26.4 °C (Step 1), (ii) from ∼ 25 to 28 °C (Step 5), (iii) from 25.9 to 27.4 °C (Step 6), (iv) from 25.2 to 30.1 °C (Step 10), and (v) from 24.4 to 27.1 °C (Step 11).

### Characterization in Organic Solvents

The final copolymers and their linear precursors (if any) were characterized in organic solvents. More specifically, their MM characteristics (number-average MM, *M*_n_, and MM distribution, MMD, given by the dispersity, *Đ*) were determined by SEC, while the compositions were calculated by NMR analysis.

### Size Exclusion Chromatography (SEC)

SEC analysis was performed in THF using an Agilent SECcurity SEC system (Agilent Technologies UK Ltd.), which is equipped with a Polymer Standard Service (PSS) SDV analytical linear M column (SDA083005LIM, dimensions: 300 × 8.00 mm, particle size: 5 μm, separation range: 0.1–1000 kg mol^–1^, an Agilent guard column (PL1110-1520, PLgel Mixed, dimensions: 50 × 7.5 mm, particle size: 5 μm), an Agilent 1250 refractive index (RI) detector, and a “1260 Iso” isocratic pump. The samples (10 mg/mL in THF) were filtered with PTFE filters with a pore diameter of 0.45 μm, and they were analyzed at a flow rate of 1 mL min^–1^. The SEC analysis was implemented by using a linear calibration curve based on six well-defined PMMA standards (the MM values are mentioned in the Materials section).

The *M*_n_ values are compared to the theoretical ones (MM^theor.^), which were calculated as the sum of the products of the MM of each monomer unit (MM_i_) multiplied by its degree of polymerization (DP_i_) and an addition of 100 g mol^–1^, which is the mass of the part of the initiator that stays on the polymer chain after the completion of dissociative GTP. Simply stated, MM^theor.^ (in g mol^–1^) is calculated by eq 1:

1 $${\mathrm{MM}}^{\mathrm{theor.}}=\underset{i}{\sum }\left({\mathrm{MM}}_{i}\times {\mathrm{DP}}_{i}\right)+100$$

### Proton Nuclear Magnetic Resonance (NMR) Spectroscopy

The NMR analysis was performed in CDCl_3_ at a concentration of ∼10 mg/mL, using a 400-MHz Avance Bruker NMR spectrometer (Bruker, UK Ltd.). For calculating the experimental compositions, the distinctive peak for BuMA and the joint peak of PEG-based methacrylate units (OEGMA300 and DEGMA) were used, which appear at 3.9 and ∼3.35 ppm, respectively. These peaks correspond to (a) the two methylene protons closest to the ester bond ((C=O)­OC**H**_2_CH_2_CH_2_CH_3_) for BuMA and the three methoxy protons at the end of the side chain (CH_2_CH_2_OC**H**_3_) for the PEG methacrylate units.

### Characterization in Aqueous Solvents

All of the copolymers were characterized in terms of their self-assembly behavior via dynamic light scattering (DLS), transmission electron microscopy (TEM), and synchrotron small-angle X-ray scattering (SAXS), which determined their hydrodynamic diameters (*d*_H_s), and morphological transitions in terms of the size and shape of the micelles. For these experiments, a concentration of 1 w/w% was used, as it represents a sufficiently low polymer concentration to minimize interparticle interactions, yet it still provides adequate scattering intensity to accurately characterize structural properties through SAXS and DLS techniques. Moreover, this concentration is above the critical micelle concentration value, which has been previously determined for ABC triblock copolymers of the same composition but slightly different MM than P7 to be in the range of 0.02 to 0.05 w/w%.^57^ In addition to these, ultraviolet-visible (UV–vis) spectroscopy, visual tests, and rheology were used to determine the cloud point temperature (*T*_CP_), gelation temperature (*T*_gel_), and gelation concentration (*C*_gel_).

### Dynamic Light Scattering (DLS)

The apparent *d*_H_s of the polymer solutions at 1 w/w% in deionized (DI) water were determined using a Zetasizer Nano ZSP (Malvern Instruments Ltd.). Prior to the experiments, the solutions were filtered using nylon filters (0.45 μm) to remove any dust or large aggregates that could interfere with the measurement. The DLS analysis was performed three times at 25 °C, and the scattered light was collected at a backscatter angle of 173°. For completion, *d*_H_s are reported as the results from both the cumulative analysis (*Z*-average) and the fitting size distributions (the latter are reported as the mean values, which correspond to the maximum of the peak by intensity and number).

### Transmission Electron Microscopy (TEM)

The self-assembled structures of the water-soluble copolymers were visualized by transmission electron microscopy (TEM). For sample preparation, 10 μL of 1 w/w% solution in DI water was pipetted onto an S160 carbon film on a 200 mesh copper grid (Agar Scientific Ltd., UK). Once 60 s has passed, the excess sample was removed using filter paper, and subsequently, the sample was washed by pipetting 30 μL of DI water. To achieve contrast on the TEM, the samples were negatively stained by using 2 w/v% uranyl acetate. The staining was performed by pipetting 30 μL of stain solution while holding the TEM grids at an angle of 45°, and the sample was left to air-dry. The TEM images were recorded using a JEOL STEM 2100Plus transmission electron microscope, operated at 80 kV to enhance contrast for bright-field TEM, and by using an objective aperture of 70 μm.

### Small-Angle X-ray Scattering (SAXS)

Synchrotron SAXS measurements of the triblock terpolymer ABC (Polymer 4) and the gradient terpolymer (Polymer 6) at 1 w/w% in H_2_O were performed at the high-brilliance beamline P12 of the European Molecular Biology Laboratory (EMBL) at the Deutsches Elektronen Synchrotron (DESY), Hamburg.^58^ X-rays with a wavelength λ = 0.155 nm were used together with a hybrid photon-counting 2D Pilatus 6M detector mounted at a sample–detector distance (SDD) of 3.0 m, which results in a *q-*range of ca. 0.02–5.8 nm^–1^. The momentum transfer is *q* = 4π sin­(θ)/λ, where 2θ is the scattering angle. The sample solution in H_2_O was loaded into an in-vacuum flow-through capillary with a 50 μm wall thickness and a 1.7 mm path length in an automatic sample changer (BioSAXS Sample Changer, Arinax), which allows loading the sample into the measurement cell, its flow through the cell during exposure as well as cleaning and drying the cell between measurements.^59,60^ Samples were measured between the two corresponding buffers for more accurate subtraction of the background arising from pure H_2_O and the empty cell. After 5 min of thermal equilibration, each polymer solution was measured at 20, 30, and 40 °C. Each sample was measured 40 times with exposure times of 95 ms. Frames that did not exhibit substantial changes due to radiation damage were averaged and azimuthally averaged using the SAXS data analysis pipeline SASFLOW^61^ within the software suite ATSAS 3.1.0.^62^

The SAXS curves were analyzed (i) by calculating the pair distance distribution function *p*(*r*) using ATSAS 3.1.0 and (ii) by fitting of structural models with the software SasView Version 5.^63^ The data from block and gradient copolymer solutions at 1 w/w% were fitted by the following model, given by eq 2:

2 $$I\left(q\right)=I_{0}P\left(q\right)S_{\mathrm{HS}}\left(q\right)+I_{\mathrm{fluc}}\left(q\right)+I_{\mathrm{bkg}}$$

where *I*_0_ is a scaling factor, *P*(*q*) is the form factor describing the shape of the micelles, and *S*_HS_(*q*) is the hard-sphere structure factor describing the spatial correlations between the micelles. *I*_fluc_(*q*) relates to local concentration fluctuations, which appear mainly in the micellar shell. *I*_bkg_ describes the background contribution.

The Percus–Yevick structure factor *S*_HS_(*q*) is used to describe the interparticle interactions through hard-sphere interactions for the ABC triblock terpolymer.^64^ It originates from the volume fraction of correlated micelles, η, and the hard-sphere radius, *R*_HS_, which is half the center-to-center distance between particles.

The Ornstein–Zernike term^65^ was used to describe the local concentration fluctuations *I*_fluc_(*q*) in the shell, giving rise to a decay of the intensity at high *q*-values. It comprises the scaling factor *I*_OZ_ and the correlation length ξ_OZ_.

The form factor of the micelles was chosen as follows: For the ABC triblock terpolymer sample, the cylinder form factor *P*_cyl_(*q*)^66^ was used for all temperatures. The model includes the cylinder cross-sectional radius *R*_cyl_, its polydispersity σ_cyl_ with a Schultz distribution, and the cylinder length *L*_cyl_, as well as the difference in the scattering length densities (SLD) of the cylinder, ρ_cyl_, and the solvent, ρ_solvent_. For the gradient terpolymer, the sphere form factor *P*_sph_(*q*)^67^ with a Schultz distribution^68,69^ of radii was used to model the SAXS curves at all temperatures, providing the radius of spheres, *R*_sph_, and the polydispersity σ_sph_.

The scattering length densities (SLDs) of OEGMA300, BuMA, DEGMA, and H_2_O were calculated as 9.71 × 10^–6^ Å^–2^, 9.85 × 10^–6^ Å^–2^, 9.42 × 10^–6^ Å^–2^, and 9.47 × 10^–6^ Å^–2^, respectively. The SLD values of the spheres and the cylinders were confined to a range of (9.42–9.85) × 10^–6^ Å^–2^, while the SLD of water was fixed at a value of 9.47 × 10^–6^ Å^–2^. In all cases, the background was limited to a range of 0.0010 ± 0.0005 cm^–1^.

### Visual Tests

The thermal changes of the aqueous polymer solutions were visually investigated by using an IKA RCT stirrer hot plate equipped with an IKA ETS-D5 temperature controller and a water bath. The polymer solutions at 1 w/w% in DI water were observed, and their *T*_CP_s were determined; *T*_CP_ is the temperature at which the solution becomes cloudy. The polymer solutions in PBS at 1, 2, 5, 10, 15, 20, and 25 w/w% were inspected, and detailed phase diagrams were constructed. The following transitions are presented in the diagrams: clear, slightly cloudy, and cloudy runny solutions; transparent and cloudy viscous solutions; and stable/gel-like solutions=the latter is operationally defined here as the absence of flow upon tube inversion for a maximum of 30 s=and macroscopic separation into two phases with gel syneresis and precipitation.

### Spectrophotometric Turbidity Evaluation

The *T*_CP_s of 1 w/w% solutions in DI water were determined by using an Agilent Cary UV–vis Compact Peltier UV–vis spectrometer with 3.5 mL cuvettes having a light path length of 10 mm. During this experiment, the solutions were heated over a temperature range close to the CP determined by visual tests. The experiment was performed at a heating rate of 1 °C min^–1^, and data were collected every 1 °C at 550 nm, which is in the middle of the visible range. The *T*_CP_ is determined as the temperature at which the transmittance reached 50%.

### Rheological Measurements

The polymer solutions at 15 and 20 w/w% in PBS were investigated using a TA Discovery HR-1 hybrid rheometer (TA Instruments, UK), equipped with a 40 mm parallel steel plate (996921), a Peltier temperature control unit, and a solvent trap; the latter was used to minimize solvent evaporation at elevated temperatures. Oscillatory temperature ramp measurements were performed from 20–80 °C, and the changes in storage modulus (or elastic modulus, *G*′ in Pa) and loss modulus (or viscous modulus, *G*″ in Pa) were recorded. The angular frequency (ω) was kept constant at 1 rad/s, while 1% strain (γ) was applied. This strain value is within the linear viscoelastic region, i.e., the area of strain values that do not disturb the network structure, as previously confirmed and reported for P7 (O_11_-*b*-B_20_-*b*-D_11_).^70^ The *T*_gel_ by rheology (if any) is determined as the temperature at which *G*′ > *G*″.

## Results and Discussion

As previously mentioned, this study explores the effect of the distribution of repeated units along the polymer chain on the self-assembly and thermoresponsive properties of copolymers based on three repeated units. First, the hydrophilic OEGMA300 (A unit) was chosen to provide water solubility, without compromising the thermogelling properties, as previously shown by our group when comparing DEGMA, OEGMA300, and oligo­(ethylene glycol) methyl ether methacrylate with an average *M*_n_ of 500 g mol^–1^.^71^ BuMA was chosen as the hydrophobic unit (B unit), as our group has previously demonstrated that BuMA-based polymers, as opposed to those with longer alkyl chains, i.e., hexyl methacrylate, produce more stable gels.^72^ To ensure thermoresponse in biologically applicable temperatures, we have incorporated DEGMA into the polymer structure an alternative to NIPAAm, which shows no hysteresis and thermoresponse at around 30 °C.^73,74^ Linear polymer structures of increasing complexity, but with constant MM, have been synthesized and are compared as follows: statistical, ABA and CBC triblock copolymers, (AC)­B­(AC), ABC, and BAC triblock terpolymers, ACBC and BABC tetrablock terpolymers, and a gradient terpolymer. These copolymers are categorized into two families: (i) copolymers with 30 w/w% BuMA content and (ii) copolymers with 35 w/w% BuMA content, and trends are compared for copolymers within the same family. These BuMA contents were targeted, as we have previously shown that the resulting copolymers possess good water solubility and exhibit interesting thermogelling properties.^28^ It is worth noting that for the gradient copolymer, the 30 w/w% BuMA content was targeted to ensure solubility in aqueous solution and allow a direct comparison with the block and statistical structures.

### Structural Properties

The successful synthesis of the polymers has been confirmed via SEC and ^1^H NMR, which provide information about the molar mass characteristics (i.e., MM and MMD) and the composition of the copolymers, respectively. The experimental structural characteristics are summarized in Table 1, where the targeted polymer structures, as well as the targeted MM values and compositions, are also presented for comparison.

**Table 1:** Theoretical Polymer Structures, Targeted Molar Masses (MM ^theor.^) and Compositions, Experimental Molar Masses (*M*_n_), and Dispersity Indices (*Đ*), as Resulted by SEC Analysis, and Experimental Compositions, as Resulted by ^1^H NMR Analysis of the Final Polymers and Their Linear Precursors (If Any)

			Molar Mass (g mol^–1^)			O-B-D (w/w%)	
No.	Distribution of Repeated Units	Theoretical Polymer Structure^a^	Theor.^b^	*M*_n_ ^c^	*Đ* ^c^	Theor.	^1^H NMR
Family A – 30 w/w% BuMA							
P1	Stat.	O_12_-*co*-B_17_-*co*-D_11_	8300	11300	1.15	45-30-25	47-29-24
P2	ABA	O_10_	2970	4200	1.13	100-00-00	100-00-00
		O_10_-*b*-B_17_	5430	7600	1.13	54-46-00	55-45-00
		O_10_-*b*-B_17_-*b*-P_10_	8300	11000	1.19	70-30-00	72-28-00
P3	CBC	D_15_	2970	4900	1.10	00-00-100	00-00-100
		D_15_-*b*-B_17_	5430	9300	1.14	54-46-00	54-46-00
		D_15_-*b*-B_17_-*b*-D_15_	8300	12500	1.23	00-30-70	00-31-69
P4^d^	ABC	O_11_	3380	5200	1.11	100-00-00	100-00-00
		O_11_-*b*-B_17_	5840	8700	1.13	57-43-00	59-41-00
		O_11_-*b*-B_17_-*b*-D_13_	8300	11500	1.18	40-30-30	41-29-30
P5	ACBC	O_12_	3790	5200	1.15	100-00-00	100-00-00
		O_12_-*b*-D_5.5_	4815	6400	1.14	78-00-22	83-00-17
		O_12_-*b*-D_5.5_-*b*-B_17_	7275	10200	1.19	51-34-14	52-34-14
		O_12_-*b*-D_5.5_-*b*-B_17_-*b*-D_5.5_	8300	11700	1.20	45-30-25	50-29-21
P6	Grad.	O_5.5_	1740	2600	1.11	100-00-00	100-00-00
		O_6.8_-*g*-B_1.4_	2356	2900	1.13	91-09-00	95-05-00
		O_8.2_-*g*-B_2.9_	2970	3500	1.12	86-14-00	93-07-00
		O_9.6_-*g*-B_4.3_	3584	3800	1.14	82-18-00	92-08-00
		O_11_-*g*-B_6_	4200	5700	1.11	80-20-00	82-18-00
		O_11_-*g*-B_12_	5020	6900	1.11	67-33-00	68-32-00
		O_11_-*g*-B_13_-*g*-D_1.6_	5534	6900	1.15	60-34-06	61-39-00
		O_11_-*g*-B_14.4_-*g*-D_3.3_	6044	7400	1.14	55-34-10	56-38-06
		O_11_-*g*-B_15.8_-*g*-D_4.9_	6556	8100	1.11	51-35-14	53-35-12
		O_11_-*g*-B_17_-*g*-D_6.5_	7070	9700	1.11	47-35-18	48-33-19
		O_11_-*g*-B_17_-*g*-D_13_	8300	11100	1.19	40-30-30	41-26-33
Family B – 35 w/w% BuMA							
P7^d^	ABC	O_11_	3380	4200	1.14	100-00-00	100-00-00
		O_11_-*b*-B_20_	6250	7700	1.16	53-47-00	55-45-00
		O_11_-*b*-B_20_-*b*-D_11_	8300	10000	1.19	40-35-25	41-34-25
P8	BAC	B_20_	2970	4200	1.11	00-100-00	00-100-00
		B_20_-*b*-O_11_	6250	8600	1.13	47-53-00	55-45-00
		B_20_-*b*-O_11_-*b*-D_11_	8300	10800	1.16	40-35-25	42-34-24
P9	(A-*co*-C)_2_B	O_5.5_-*co*-D_5.5_	2765	4100	1.14	62-00-38	66-00-34
		(O_5.5_-*co*-D_5.5_)-*b*-B_20_	5635	8900	1.14	30-52-18	34-51-15
		(O_5.5_-*co*-D_5.5_)-*b*-B_20_-*b*-(O_5.5_-*co*-D_5.5_)	8300	12800	1.18	40-35-25	43-36-21
P10	BABC	B_10_	1535	2100	1.15	00-100-00	00-100-00
		B_10_-*b*-O_11_	4815	6100	1.14	70-30-00	70-30-00
		B_10_-*b*-O_11_-*b*-B_10_	6250	8000	1.21	53-47-00	56-44-00
		B_10_-*b*-O_11_-*b*-B_10_-*b*-D_11_	8300	10700	1.14	40-35-25	44-34-22

a O, B, and D are the one-letter abbreviations for oligo­(ethylene glycol) methyl ether methacrylate with an average Mn of 300 g mol^–1^, *n*-butyl methacrylate, and di­(ethylene glycol) methyl ether methacrylate, respectively.

b The theoretical MM is calculated using the eq 1: MM^theor.^(gmol^–1^) = (∑*_i_* MM*_i_* × DP*_i_*) + 100; 100 g mol^–1^ is the MM of the MTS part that stays on the polymer chain after the completion of dissociative GTP.

c The number-average molar mass (*M*_n_) and the dispersity (*Đ*) of the polymers and their precursors (if any) were determined by size exclusion chromatography (SEC). The calibration curve was plotted as a linear function of the MM versus the elution time of well-defined linear poly­(methyl methacrylate) (PMMA) standard samples with the following MM values: 2000, 4000, 8000, 20000, 50000, and 100000 g mol^–1^.

d The synthesis and investigation of the ABC triblock terpolymers were previously published by our group, but they are also included here for the completion of the study and direct comparison.^28,70^

As can be seen in Table 1, the *M*_n_ values vary between 10000 and 12800 g mol^–1^, which is satisfactorily close to the target MM (8300 g mol^–1^). This discrepancy may be attributed to the SEC analysis being relative, i.e., based on the use of PMMA polymers as calibration standard samples.

As the MMD is crucial in controlling the final properties, it is important that well-defined polymers are synthesized. The polymers presented in this study are not only of controlled MM, but their MMD is also controlled, as their *Đ* values vary between 1.11 and 1.23. This observation confirms the successful “living” GTP.

Of major interest is the synthesis of the gradient copolymer, the SEC traces of which are presented in Figure 3a. As can be seen, the synthesis is successful as the polymerization progresses from OEGMA300_5.5_ (Sample 1, in light blue solid line), to OEGMA300_11_-*g*-BuMA_6_ (Sample 5, in orange solid line), to OEGMA300_11_-*g*-BuMA_12_ (Sample 6, in red solid line), to OEGMA300_11_-*g*-BuMA_17_-*g*-DEGMA_6.5_ (Sample 10, in blue solid line), and to OEGMA300_11_-*g*-BuMA_17_-*g*-DEGMA_13_ (Sample 11, in dark blue solid line). The SEC traces shown in dashed and dotted lines were obtained during the monomer addition, as explained in the experimental section and presented in Figure 2. The SEC traces of the final gradient terpolymer show a unimodal distribution with slight tailing toward the low MM values, which indicates only slight deactivation originating from the previous synthetic steps, thus confirming the successful sequential GTP. This is also confirmed by its low MMD (*Đ* < 1.2). The SEC traces of the rest of the polymers and their precursors (if any) are presented in Figures S1 and S2, and they show similar trends.

**Figure 3:**
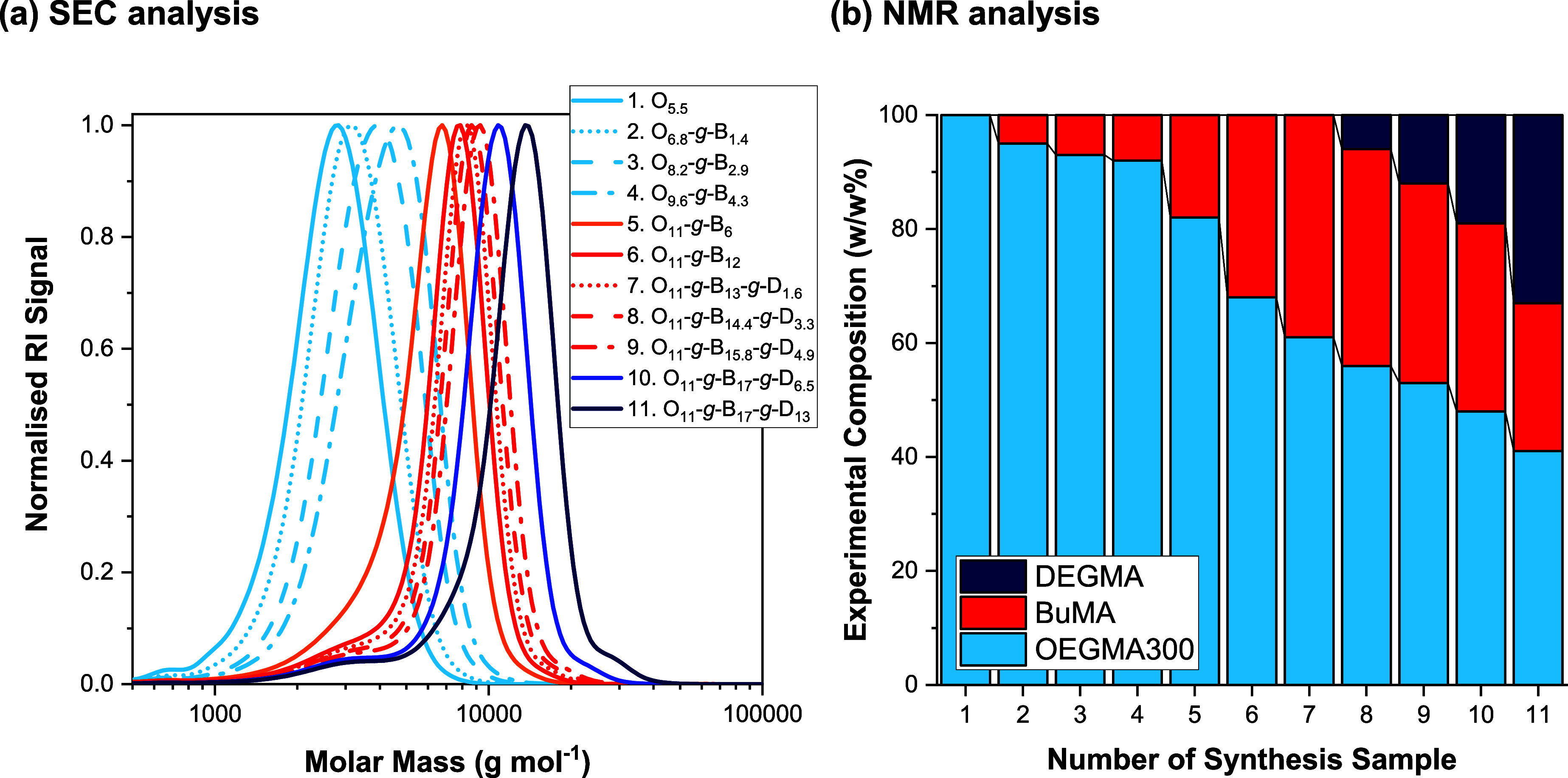
(a) SEC traces resulting from the synthesis of the gradient terpolymer (Polymer 6). The traces shown in solid lines correspond to the samples obtained after complete consumption of the monomer, while the chromatograms presented in dotted, dashed, and dotted-dashed lines were obtained during the GTP synthesis. (b) Variation of the experimental composition, resulted by NMR analysis, of OEGMA300, BuMA, and DEGMA in the gradient terpolymer (Polymer 6) as the GTP progresses shown in light blue, red, and dark blue, respectively. Note: readers may refer to Figure 2 and Table 1 for more information.

The experimental compositions are satisfactorily close to the targeted ones, within the error of the NMR technique (Table 1). Figure S3 shows the NMR spectra of the gradient terpolymer and its precursors. As can be seen, in each synthetic step, the additional peaks expected are observed, and all of the peaks corresponding to all three monomers are detected in the final terpolymer. Interestingly, the NMR analysis of the gradient terpolymer and its precursors confirms the gradient change in composition as the polymerization progresses (Figure 3b). As evidenced, in the first synthetic steps, the % composition of OEGMA300 decreases, while the BuMA content increases, thus indicating the descending and ascending gradients in OEGMA300 and BuMA content. As the synthesis progresses further, DEGMA is added to the system; thus, the content of both OEGMA300 and BuMA gradually decreases, while an ascending gradient in DEGMA content is observed.

### Aqueous Solution Properties

#### Hydrodynamic Diameters

When amphiphilic block copolymers are dissolved in aqueous media, they self-assemble into micelles, with the hydrophobic block forming the core of the micelle, thus avoiding interactions with water, while the shell consists of the hydrophilic block that interacts with water molecules. On the other hand, polymers with a random or statistical distribution of repeated units within the polymer chain cannot form any ordered self-assembled structures because they do not consist of distinct hydrophobic and hydrophilic parts. Thus, it is expected that the statistical terpolymer, if water-soluble, will adopt a random coil configuration, while the block copolymers will self-assemble into micelles. In addition, gradient copolymers have been shown experimentally to form micelles in solution depending on temperature.^75,76^ Besides, the self-assembly behavior was also addressed computationally in recent studies.^77,78^ More specifically, in the current study, it is expected that the self-assembly is driven by the hydrophobicity of the BuMA blocks while exposing the hydrophilic OEGMA300 and DEGMA units.

Prior to any physicochemical characterization, the solubility of the polymers in aqueous media was investigated. The statistical terpolymer (Polymer 1) is not soluble due to its inability to form micelles. The CBC triblock copolymer (Polymer 3) is also not soluble, presumably because of the increased hydrophobicity of the structure, caused by the absence of the hydrophilic OEGMA300 units, in combination with the low LCST of DEGMA (26 °C, depending on MM).^79^ This indicates the necessity of incorporating OEGMA300 into the structure to promote solubility. In addition to the statistical and CBC copolymers, the ACBC copolymer (Polymer 5) is also insoluble, in agreement with previously reported ACBC tetrablock terpolymers.^21^

To probe the size of the self-assembled nanostructures at room temperature, DLS analysis was implemented (Table 2 and Figures S4–S7). As can be seen, monomodal distributions are detected, with the sizes of the aggregates varying based on the distribution of repeated units and composition. In general, larger self-assembled nanostructures are observed for the copolymers with 35 w/w%, except for the BABC copolymer (with 35 w/w% BuMA), which forms the smallest micelles, as revealed by DLS. Despite the advantage of DLS being a widely used and easily accessible characterization technique, the DLS-based estimation of hydrodynamic diameter distributions assumes diffusing spherical species, which may not be accurate depending on the distribution of repeated units within the polymer structure, as will be discussed in the SAXS section.

**Table 2:** Theoretical Polymer Structures, Distribution of Repeated Units, Experimental Hydrodynamic Diameters (*d*_h_), and Polydispersity Indices (PDIs) of 1% (w/w) Polymer Solutions in Deionized (DI) Water at 25 °C^d^

			*d*_h_ (nm) Exp. ± 0.5^b^			
No.	Distribution of Repeated Units	Theoretical Structure^a^	Z-average	By Intensity	By Number	PDI
Family A – 30 w/w% BuMA						
P1	Stat.	O_12_-*co*-B_17_-*co*-D_11_	NS			
P2	ABA	O_10_-*b*-B_17_-*b*-P_10_	22.9	24.4	11.7	0.130
P3	CBC	D_15_-*b*-B_17_-*b*-D_15_	NS			
P4^c^	ABC	O_11_-*b*-B_17_-*b*-D_13_	20.2	21.0	11.7	0.117
P5	ACBC	O_12_-*b*-D_5.5_-*b*-B_17_-*b*-D_5.5_	NS			
P6	Grad.	O_11_-*g*-B_17_-*g*-D_13_	16.9	18.2	11.7	0.034
Family B – 35 w/w% BuMA						
P7^c^	ABC	O_11_-*b*-B_20_-*b*-D_11_	44.4	43.8	21.0	0.219
P8	BAC	B_20_-*b*-O_11_-*b*-D_11_	51.6	50.7	21.0	0.228
P9	(AC)_2_B	(O_5.5_-*co*-D_5.5_)-*b*-B_20_-*b*-(O_5.5_-*co*-D_5.5_)	33.8	37.8	18.2	0.158
P10	BABC	B_10_-*b*-O_11_-*b*-B_10_-*b*-D_11_	13.3	13.5	8.7	0.077

a Abbreviations: di- and oligo­(ethylene glycol) methyl ether methacrylate with an average *M*_n_ of 300 g mol^–1^ (DEGMA/D and OEGMA300/O), *n*-butyl methacrylate (BuMA/B).

b The experimental *d*_h_ values are the mean diameters which correspond to the maximum of the peak by intensity and by number.

c The results are also presented in our recent publication on the effect of composition, but they are also included here for the completion of the study and direct comparison.^28,70^

d Not soluble (NS): the polymers were not soluble in DI water, and thus, their characterization in aqueous solutions was not feasible.

#### Transmission Electron Microscopy Images

The diluted aqueous solutions of the water-soluble copolymers (1 w/w% in deionized water) were imaged via TEM (Figure 4). The TEM images reveal the formation of nearly spherical micelles of various sizes, which depend on the polymer structure. Evidently, the smallest self-assembled structures, around 10 nm, are formed by the BABC copolymers, in high agreement with the DLS results, according to which it forms micelles with monomodal size distributions peaking at 13.5 nm in intensity and 8.7 nm by number. Concerning the polymers with 30 w/w% BuMA, namely, Polymer 2 (ABA), Polymer 4 (ABC), and Polymer 6 (gradient), they all form micelles around 20 nm, also in agreement with the DLS results. The results of Polymer 9 ((A-*co*-C)-*b*-B-*b*-(A-*co*-C)) reveal spherical structures of around 25 nm, which are located close to each other, nearly merging, thus forming structures of around 45 nm in size. These results also broadly agree with the DLS results, according to which *d*_h_ values of 37.8 and 18.2 nm were detected by intensity and by number, respectively. On the other hand, structures of varying sizes between 14 and 25 nm were captured for Polymer 7 (ABC) and Polymer 8 (BAC), which are smaller than the sizes detected by DLS. This may be attributed to the different experimental conditions, as the DLS is performed in the aqueous state and reports the hydrodynamic sizes for diffusing spheres, while the TEM is performed in the dry state and in the presence of uranyl acetate, which might slightly interfere with the structure, as previously observed and reported.^80^

**Figure 4:**
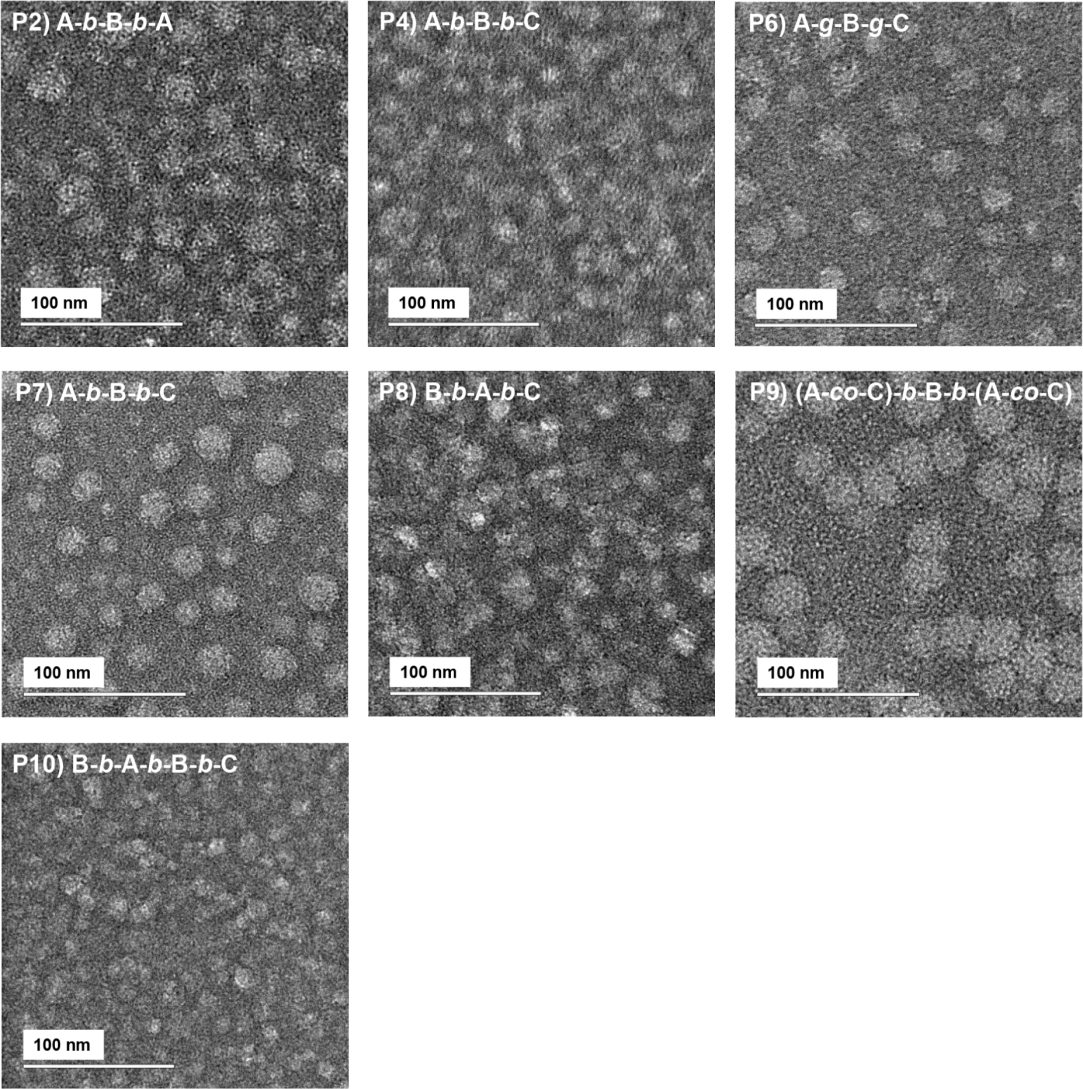
Transmission electron microscopy (TEM) images of 1% w/w polymer solutions in deionized water, negatively stained with uranyl acetate.

#### Cloud Point Temperatures

The diluted aqueous polymer solutions (1 w/w%) were investigated for thermal response, and their *T*_CP_s by visual tests and UV–vis are shown in Figure 5 with squares and rhombi, respectively; the relevant curves of transmittance versus temperature are shown in Figure S8.

**Figure 5:**
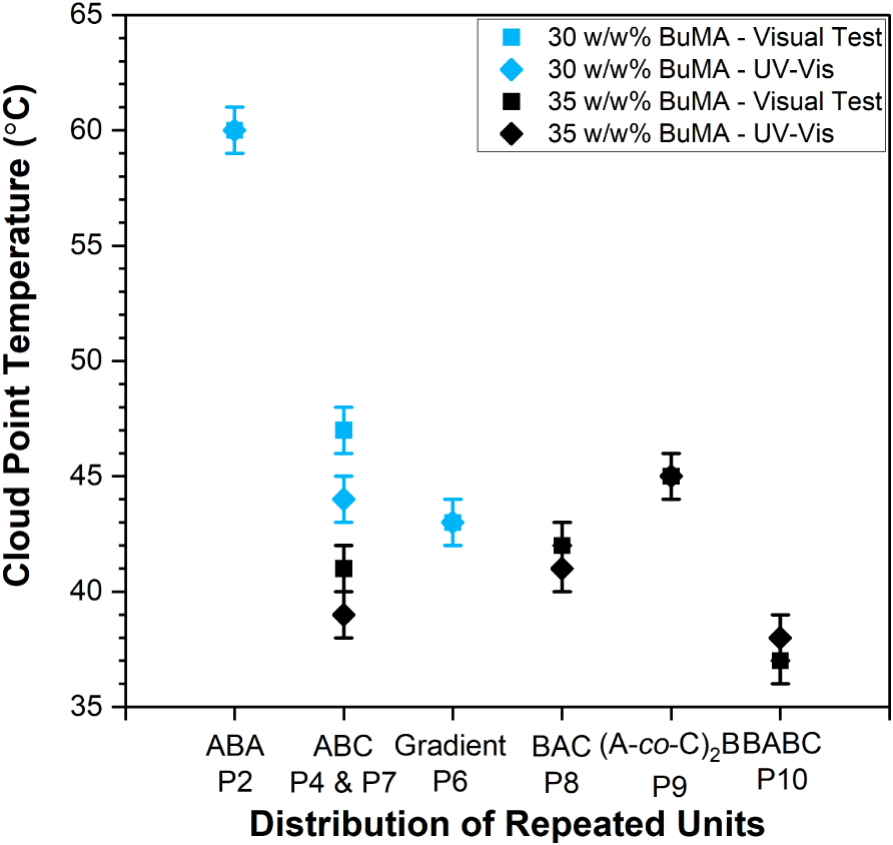
Effect of the distribution of repeated units on the cloud point temperature (*T*_CP_), as determined by visual tests (squares) and UV–vis spectroscopy (rhombi). The *T*_CP_ of the copolymers with 30 w/w% BuMA content is shown in light blue, while the *T*_CP_ of the copolymers with 35 w/w% BuMA is shown in black.

The effect of the distribution of repeated units within the polymer chain and, complementarily, the effect of composition are shown in Figure 5; the *T*_CP_s of the copolymers with 30 and 35 w/w% BuMA content are shown in light blue and black, respectively. As is evident, the ABA triblock copolymer (Polymer 2) presents the highest *T*_CP_, because of its higher hydrophilicity, caused by the absence of DEGMA units. This can be explained by considering the *T*_CP_s of OEGMA300 and DEGMA homopolymers, which are around 64 °C and 26 °C, MM-dependent.^79^ Therefore, it is concluded that the absence of DEGMA increases the *T*_CP_, while the incorporation of BuMA lowers the *T*_CP_, as it enhances the “hydrophobic effect”, i.e., the entropic contributions by the release of water molecules. Regarding the DEGMA-containing polymers, they present a *T*_CP_ between 37 and 47 °C, in agreement with previous studies on DEGMA-based copolymers.^21–28^

#### Micellar Structures – Synchrotron SAXS

As will be discussed below, ABC triblock and gradient copolymers presented the most interesting gelation profiles. Therefore, we utilized synchrotron SAXS to investigate the micellar structures of Polymer 4 (ABC triblock terpolymer) and Polymer 6 (ABC gradient terpolymer) in dilute aqueous solution (1 w/w%). These two terpolymers were chosen because they have the same composition and overall degree of polymerization. We investigated the structures as a function of temperature between 20 and 40 °C, i.e., below their respective cloud point temperatures *T*_CP_, which are 47 and 43 °C for the triblock and gradient terpolymers, respectively (Figure 5). The SAXS curves are displayed in Figure 6. The deconvolutions and all fitting parameters are given in Figures S9 and S10 and in Tables S1 and S2.

**Figure 6:**
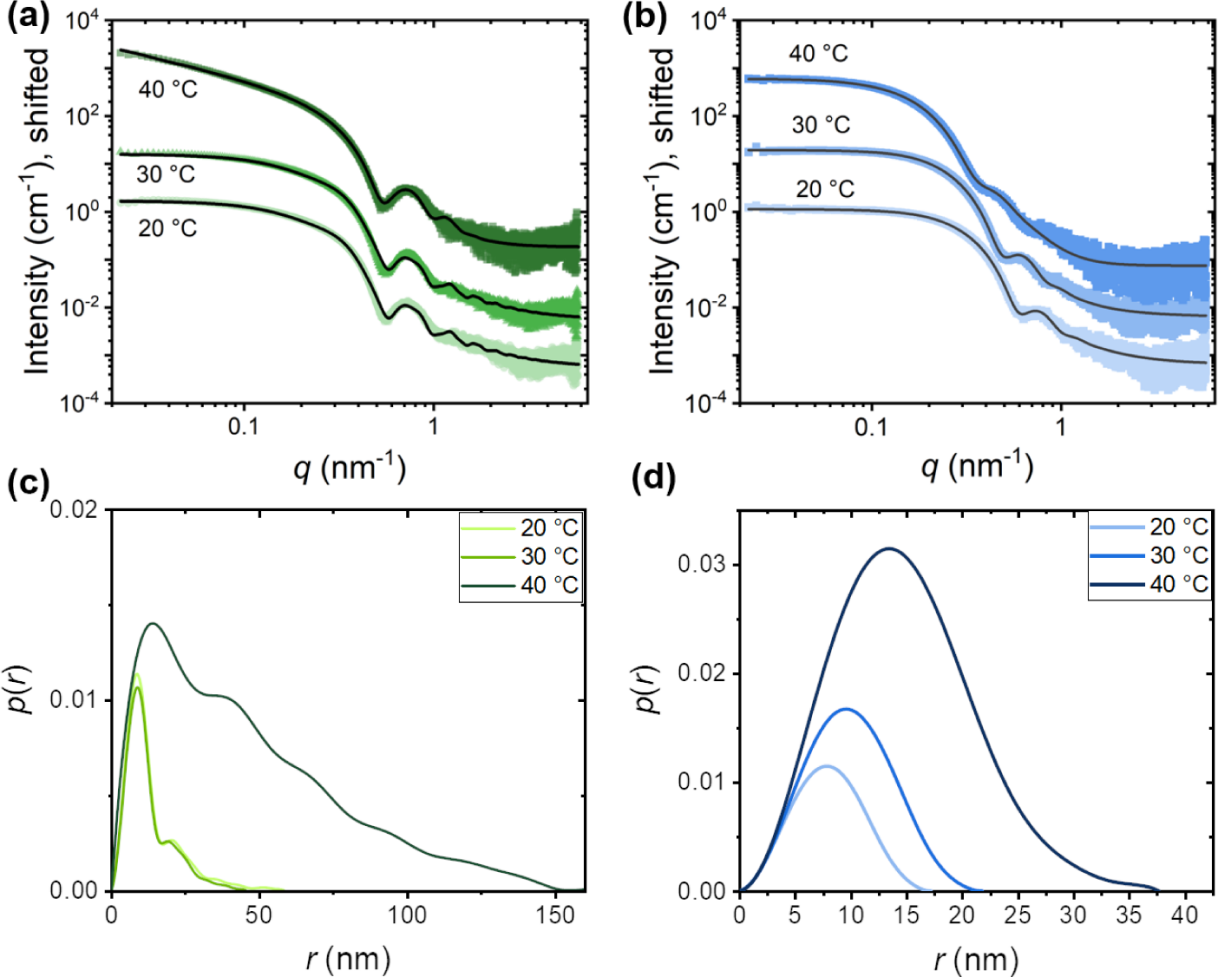
SAXS curves of 1 w/w% solutions of (a) the ABC triblock terpolymer (Polymer 4) and (b) the gradient terpolymer (Polymer 6) in deionized water at various temperatures. The symbols show the experimental data, while the solid lines are model fits; see text. The curves at 30 and 40 °C are shifted upward by factors of 10 and 100, respectively. Pair distance distribution function *p*(*r*) of (c) the ABC triblock terpolymer and (d) the gradient terpolymer in in deionized water at various temperatures.

For the ABC triblock terpolymer (Figure 6a), the curves at 20 and 30 °C have similar shapes, featuring a plateau up to ca. 0.3 nm^–1^, a shoulder, a fringe at 0.7 nm^–1^, and a weak decay above. At 40 °C, instead of a plateau at low *q*-values, the SAXS curve exhibits a decay with a slope between −1 and −2 in the double-logarithmic representation, indicating the formation of elongated particles. The model-free pair distance distribution function *p*(*r*) supports this hypothesis for the ABC triblock terpolymer sample at 20 and 30 °C (Figure 6c), which exhibits asymmetrical distributions with a clear maximum at relatively small distances corresponding to the cross-sectional radius of the long particles. At 40 °C, the maximum of the *p*(*r*) curve and *r*_max_ shift to the right with the appearance of fringes, suggesting an increase of the cross-sectional radius of the elongated particles, as well as a more compact structure. In contrast, the SAXS curves of the gradient terpolymer (Figure 6b) feature a plateau at low *q*-values at all temperatures, and with increasing temperature, the shoulder and the fringe move to lower *q*-values. The *p*(*r*) curves (Figure 6d) display a bell shape at 20 °C and maintain the same shape with increasing temperatures. In addition, the peak of the curve shifts to higher *r* values upon heating, which indicates a size increase without a shape change.

At 20 °C, the SAXS data from the ABC triblock terpolymer can be fitted by the model given in eq 2, in which, for the ABC triblock terpolymer sample, *P*(*q*) was chosen to be the form factor for homogeneous cylinders. We expected that below *T*_CP_, we would observe a core–shell structure with the hydrophobic B block constituting the core and the hydrophilic A block and the thermoresponsive C block forming the shell; however, the expected core–shell structure of micelles, could not be resolved by SAXS, as found in preliminary fits. As shown in Figure 7a, at 20 and 30 °C, the resulting cross-sectional radius *R*_cyl_ is ca. 7 nm with a very narrow dispersity, and the length of the cylinders, *L*_cyl_, is ca. 34 nm. These values are compatible with the hydrodynamic diameter of 21.0 nm found by DLS at 25 °C (Table 2). We speculate that *R*_cyl_ mainly reflects the dense BuMA core together with a certain inner part of the shell, where the shell blocks are also rather densely packed. It was necessary to include the Ornstein–Zernike structure factor with correlation lengths ξ_OZ_ of 1.3 and 1.5 nm at 20 and 30 °C; that is, the part of the shell that is observed is hydrated. Moreover, we hypothesize that these micelles are in reality spherical, as demonstrated by TEM (Figure 4) and that 2–3 micelles have associated to form short cylinders. Such a mechanism—formation of a hydrophobic channel by association of the hydrophobic cores—was discussed previously.^81^ This is reflected by the need to include a structure factor, which gives a hard-sphere radius *R*_HS_ of 7.3 nm at 20 °C and 7.1 nm at 30 °C. These values might correspond to the half-distances between the spherical micelles.

**Figure 7:**
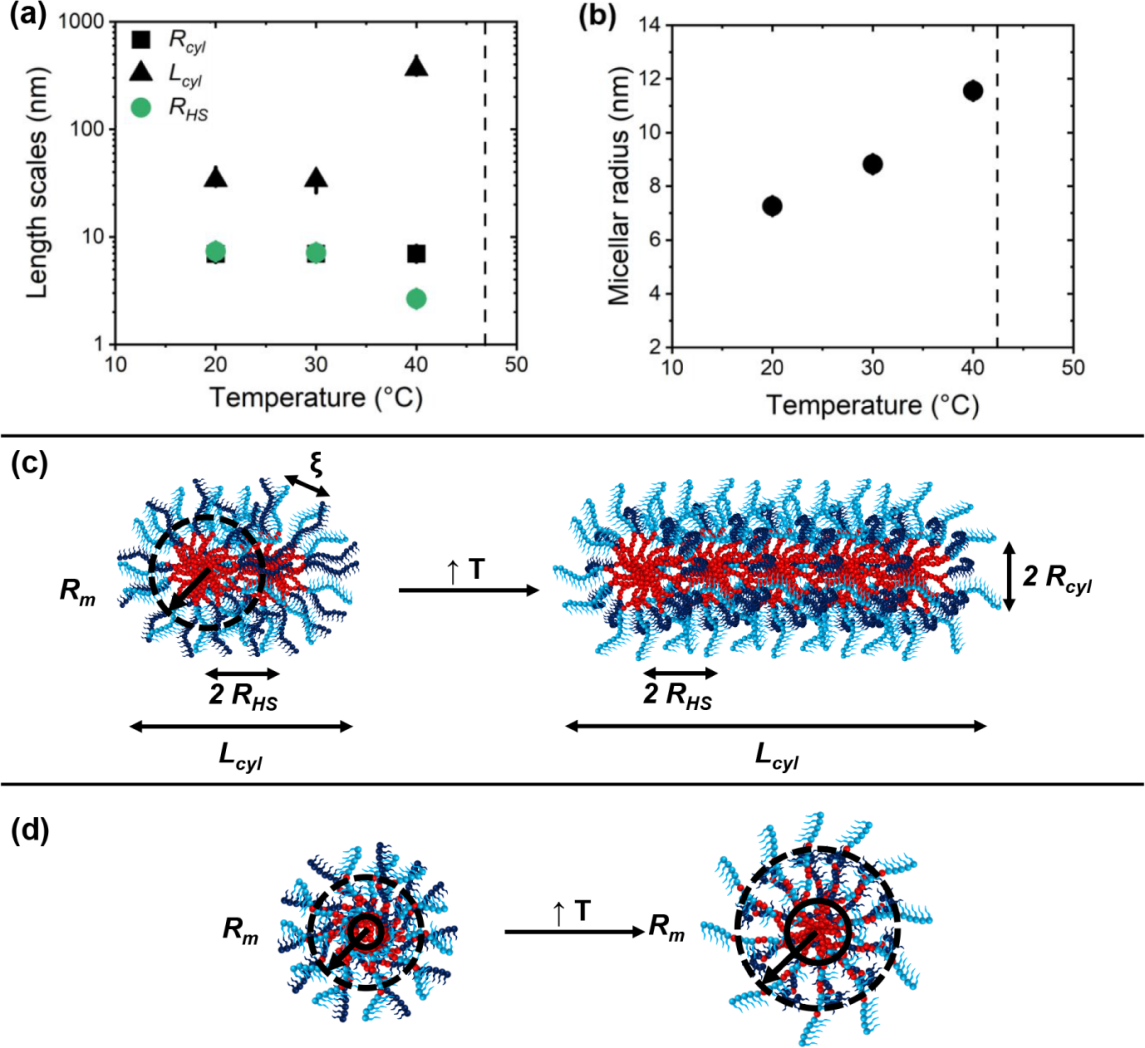
Structural parameters resulting from the fits shown in Figure 6 as a function of temperature. (a) ABC triblock terpolymer: cross-sectional radius *R*_cyl_ (black squares), cylinder length *L*_cyl_ (black triangles), and hard-sphere radius *R*_HS_ (green circles) in a semilogarithmic representation. (b) ABC gradient terpolymer: micellar radius *R*_m_. The black dashed lines indicate the *T*_CP_ values from Figure 5. The resulting temperature-dependent structures for the ABC triblock terpolymer and ABC gradient terpolymer are schematically shown in parts (c) and (d), respectively, along with a few structural parameters.

At 40 °C, for the triblock terpolymer solution, *R*_cyl_ is still 7 nm, but *L*_cyl_ has increased significantly, namely to 360 nm, which is the origin of the decay of the SAXS curve at low *q*-values. Moreover, *R*_HS_ decreased strongly to 2.7 nm; however, the volume fraction of correlated micelles is very low. The Ornstein–Zernike structure factor was not needed, suggesting that the inner part of the shell is relatively compact due to the contraction of the thermoresponsive PDEGMA blocks close to *T*_CP_. We conclude that, at 40 °C, a large number of spherical micelles form a long cylinder and interpenetrate strongly, as suggested by the low value of *R*_HS_ compared to *R*_cyl_, which may be due to the contraction of the PDEGMA blocks. The findings are schematically shown in Figure 7a.

The gradient terpolymers self-assemble into seemingly homogeneous, spherical micelles, which grow steadily with increasing temperature, do not change shape, and the SAXS curves were fitted employing a spherical form factor. Their radius *R*_m_ increases steadily from 7.3 nm at 20 °C to 8.8 nm at 30 °C and further to 11.6 nm at 40 °C (Figure 7b) with the width of the distribution slightly increasing (Table S2). The values at 20 and 30 °C are compatible with the hydrodynamic diameter of 18.2 nm found by DLS at 25 °C (Table 2). They are slightly larger than *R*_cyl_ of the triblock terpolymer, which may be attributed to a larger inner dense part of the micelles, possibly due to the dispersed B segments in the A and C shell blocks. The increase in micellar size with temperature might be attributed to an increase of the size of a single micelle, but this does not seem probable given the expectation that the C segments contract upon heating. We hypothesize that, upon heating, the C segments contract and merge with the B core. In doing so, they drag more B segments into the core, which results in an increase of *R*_m_. For the description of the SAXS curves of the gradient terpolymer solution, no structure factor was needed; i.e., the micelles do not appear to interact with each other. The formation of a hydrophobic channel does not seem to be necessary. Possibly, when the C blocks contract and approach the core, the outer part of the shell mainly consists of hydrophilic A blocks, which stabilize the single micelles.

Comparing the micellar structures of the triblock terpolymer with those of the gradient terpolymer, we observe different micellar shapes (cylinders vs spheres) and different growth behavior (strong increase of cylinder length at a constant cross-sectional radius vs steady growth), in spite of the same composition. The cylinders formed by the triblock terpolymer possibly contain several spherical micelles that have associated and formed a hydrophobic channel, which is promoted as *T*_CP_ is approached and the C blocks contract. In contrast, the micelles from the gradient terpolymers stay dispersed and are stabilized by the A blocks that extend outward when the C segments contract. These differences at the level of dilute solutions of micelles may be the origin of the different gelation behavior.

#### Gel Points – Phase Diagrams

To test thermogelation under physiologically relevant conditions, homogeneous polymer solutions in PBS were evaluated over a range of temperatures, and detailed phase diagrams consisting of the following transitions were constructed and are presented in Figure 8: (i) runny solution phase, represented by white symbols, with squares, triangles, and circles indicating clear, slightly cloudy, and cloudy solutions, respectively; (ii) viscous solution phase, represented by red, with triangles and circles indicating transparency and cloudiness, respectively; (iii) stable gel-like state, represented by blue , with triangles and circles showing transparency and cloudiness, respectively; and (iv) two phases, represented by green, indicating either gel syneresis (in rhombi) or precipitation (in squares). In Figure 8, seven phase diagrams are presented, as the three remaining polymers are not soluble in aqueous solvents, as previously. Interestingly, four copolymers present a gelation area, indicated by a black dashed line, while only two polymers do not form gels at any temperature or concentration tested.

**Figure 8:**
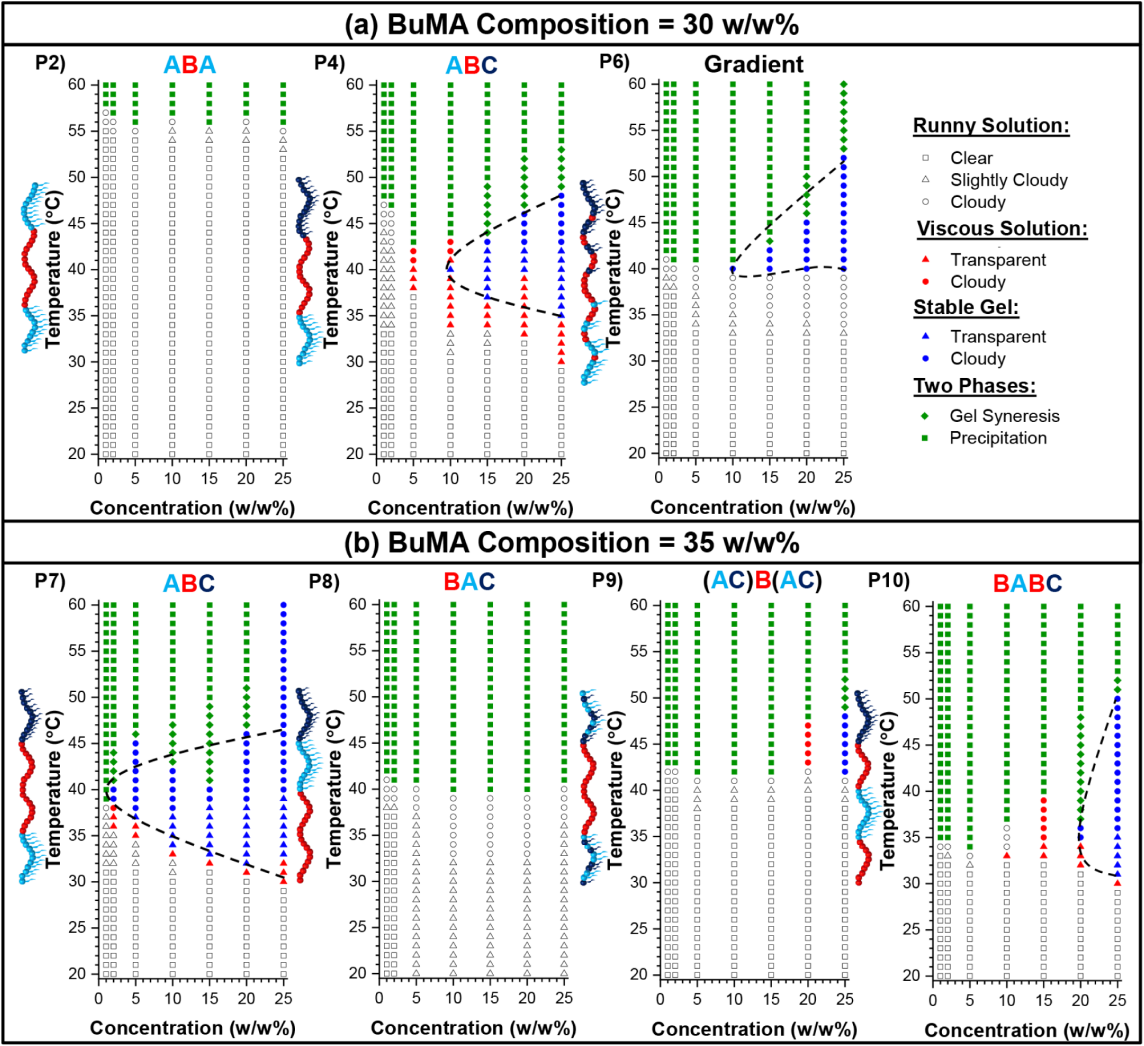
Phase diagrams in phosphate-buffered saline (PBS) of all the soluble copolymers with BuMA composition equal to (a) 30 w/w% (top) and (b) 35 w/w% (bottom). The transitions are shown in white for runny solutions (squares: clear, triangles: slightly cloudy, circles: cloudy), red and blue for viscous solutions and stable gels, respectively (triangles: transparent, circles: cloudy), and gel syneresis and precipitation in green rhombi and squares, respectively. The gel area is approximately shown in a black dashed line. * The phase diagrams of Polymers 4 and 7 have been previously published, but they are included here for completion.^28,70^

Among the polymers that do not form stable gels upon temperature increase are the ABA triblock copolymer (Polymer 2) and the BAC triblock terpolymer (Polymer 8). More specifically, they both present a *T*_CP_ at elevated temperatures, followed by phase separation. When these two copolymers are compared, the BAC triblock copolymer clearly responds at lower temperatures than the ABA triblock copolymer. Notably, the ABA triblock copolymer presents thermoresponse at a higher temperature range than the terpolymers, thus indicating the importance of incorporating the DEGMA unit to present thermoresponse close to biological conditions. The inability of the BAC structure to form gels agrees with previous studies on DMAEMA polymers.^82^

Among the best-performing linear architectures in terms of gelation area are the ABC gradient terpolymer and the ABC and BABC block copolymers, while the (AC)­B­(AC) triblock terpolymer only forms a gel at sufficiently high concentrations. The reduced ability of the (AC)­B­(AC) copolymer to form gels may be attributed to the DEGMA and OEGMA300 units being randomly distributed within the hydrophilic corona, thus disrupting the sufficient formation of physical cross-links. The results regarding the ABC and BABC copolymers are comparable to our previous findings on DMAEMA-containing polymers.^83^ When the gradient copolymer is compared to the corresponding ABC triblock terpolymers, it is concluded that the gelation area is slightly shifted to higher temperature or concentration ranges. These differences may be attributed to the temperature-dependent structural changes between the gradient- and block-based micelles. Specifically, as previously mentioned, the ABC triblock copolymer micelles grow into elongated cylindrical structures with temperature, while the ABC gradient copolymer micelles grow but remain spherical. Therefore, one could speculate that the formation of cylindrical channels may favor gelation at lower temperatures and concentrations. Despite the shift in the gelation area of the gradient copolymer, this region could be tuned to physiologically relevant conditions by modifications in its MM and composition. Thus, the gradient structure opens new possibilities for identifying thermoresponsive polymers that can serve either as injectable gels or 3D printable materials.

### Gel Points – Rheology

The soluble copolymer solutions at 15 and 20 w/w% in PBS were investigated via rheology, and their gel points (if any) were determined and compared to the results of visual tests. At this point, it should be noted that a gel point by rheology is defined as the point, either temperature or time, at which the storage modulus (or elastic modulus, *G*′) exceeds the loss modulus (or viscous modulus, *G*″). The graphs showing the variation in storage and loss moduli as a function of temperature are presented in Figure 9. The structures are also schematically illustrated, with light blue, red, and dark blue representing the units of OEGMA300, BuMA, and DEGMA, respectively.

**Figure 9:**
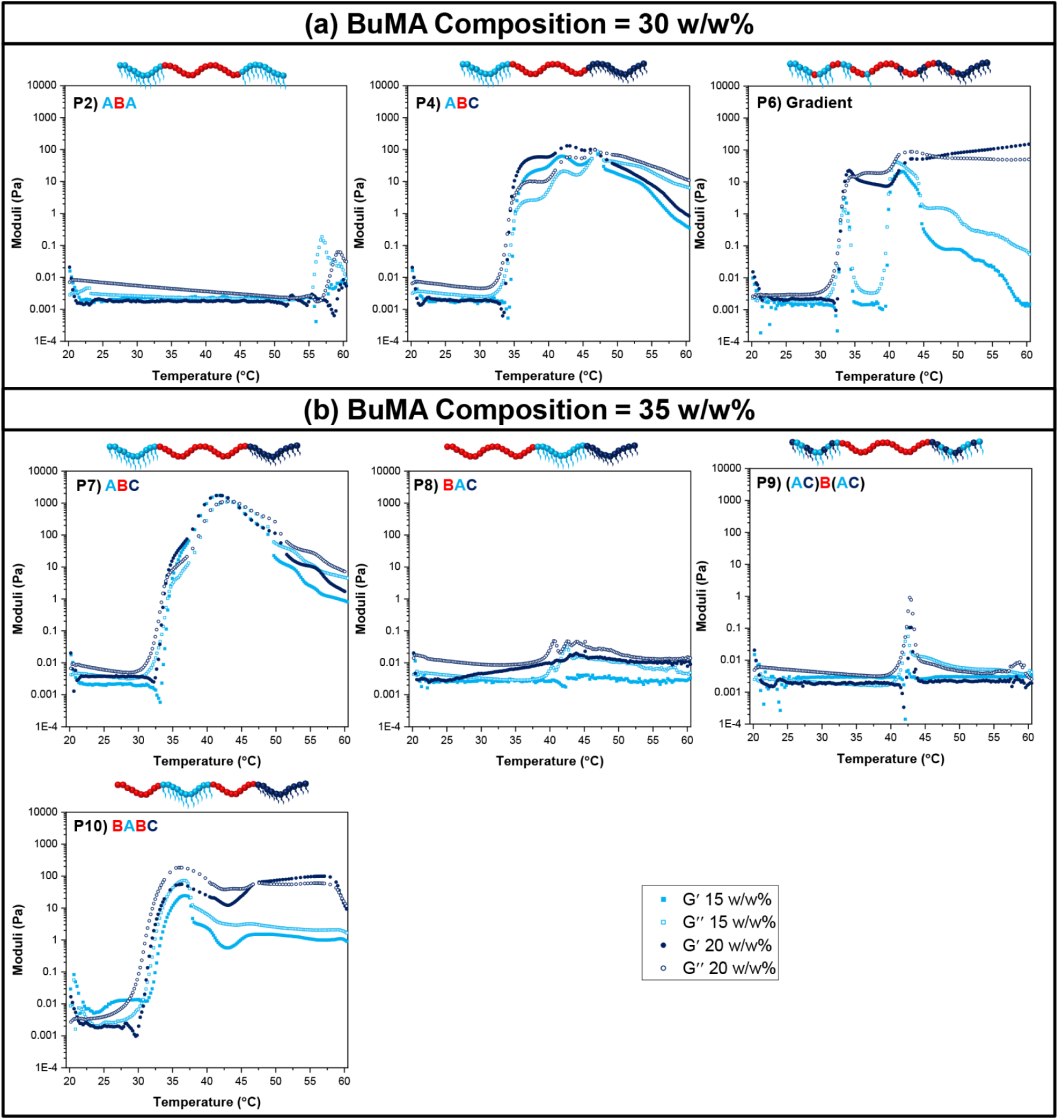
Rheological curves of the soluble copolymers at 15 w/w% (light blue squares) and 20 w/w% (dark blue circles) in phosphate-buffered saline (PBS). The changes in storage (*G*′) and loss (*G*″) moduli are shown as filled and open symbols, respectively.

The ABA (Polymer 2), BAC (Polymer 8), and (AC)­B (AC) (Polymer 9) block copolymers do not form gels at either concentration according to rheology, which is highly consistent with the visual observations reported in Figure 8. Interestingly, a nonmonotonous behavior is observed as a local maximum at temperatures where the samples visually exhibit macroscopic phase separation in the form of a cloud point, followed by precipitation.

Regarding the ABC triblock terpolymers (Polymers 4 and 7), the moduli increase, and eventually, the storage modulus exceeds the loss modulus, indicating the likely formation of a gel, with Polymer 7 forming a stronger gel than Polymer 4, attributable to its higher hydrophobic content. These observations agree with the results from visual tests—see Figure 8—in which gelation was observed within a similar temperature range at these concentrations. Regarding the temperatures at which the gels destabilize, these are detected by rheology at temperature values between gel syneresis and precipitation. This agrees with our previous observations^28^ and is attributed to the syneresis being subtle and thus not detectable by rheology.

The gradient terpolymer (Polymer 6) and the BABC block terpolymer (Polymer 10) present interesting rheological profiles. Regarding the gradient terpolymer, a narrow gelation window is visually detected by the tube inversion method at around 40 °C, followed by gel syneresis and macroscopic phase separation into liquid solvent and precipitated polymer. Interestingly, an initial peak at around 33 °C is detected by rheology, which matches the transition temperature from a clear to a cloudy solution, followed by a secondary transition at 40 °C, where gelation is visually detected. On the other hand, BABC solutions do not form a hydrogel at 15 w/w% and only present a narrow gelation window at 20 w/w% in PBS. These transitions are detected as an increase in viscosity by rheology. These differences are attributed to the limited stability of the gels and the different nature of the techniques, i.e., tube inversion versus application of shear stress.

In general, when the polymer solutions transition from the liquid phase to phase separation, this is detected as a low-intensity peak by rheology. On the other hand, a visual increase in viscosity and or gelation is confirmed by rheological measurements. Therefore, it can be concluded that both techniques, i.e., visual inspection via the tube-inversion method and rheology, provide complementary information on the behavior of concentrated thermoresponsive polymer solutions.

## Conclusions

In this study, several polymers with increasing complexity in the distribution of repeated units have been studied and are reported. All the copolymers were successfully synthesized, as confirmed by SEC and ^1^H NMR. Interestingly, a clear effect of the distribution of repeated units in copolymers based on OEGMA300, BuMA, and DEGMA on their properties is reported for the first time. The necessity of incorporating the OEGMA300 units is demonstrated by the CBC triblock copolymer being insoluble, while the importance of incorporating the DEGMA units is confirmed by the inability of the ABA structure to respond near biological temperature or form gels. Concerning the terpolymers, it is observed that the statistical and ACBC tetrablock terpolymers are insoluble in aqueous media, while the rest of the terpolymers are soluble in aqueous media, presenting an interesting self-assembly behavior and thermoresponse. Specifically, the micellar shapes and the aggregation mechanisms differ strongly between the triblock and the gradient terpolymer. Among the best-performing structures, i.e., copolymers whose solutions form gels upon temperature increase, are the ABC triblock structure and, notably, the gradient terpolymer structure. In dilute solution, the ABC triblock terpolymers self-assemble into short cylinders that become longer upon heating toward *T*_CP_, presumably due to the formation of hydrophobic channels between the spherical core–shell micelles, which is favored by the contraction of the C blocks as *T*_CP_ is approached. In contrast, the gradient terpolymer forms spheres that grow as the temperature approaches *T*_CP_, without correlation between micelles. We assign this counterintuitive finding to the growth of the inner part of the micelle which grows when the C segments contract and merge with the B core, while dragging B segments from the gradient region along. Thus, the packing of the terpolymers within the micelles and their association behavior differs between the triblock and gradient structures. These observations open a new horizon in the field of gradient terpolymers, as slight modifications in their structure might reveal new thermoresponsive polymers that are biologically applicable.

## Supplementary Material



## Notes

SEC chromatograms of the copolymers and their precursors (if any); ^1^H NMR spectra of the gradient terpolymer and its precursors; DLS histograms by intensity and by number obtained by analyzing solutions 1 w/w% in deionized water at 25 °C; transmittance as a function of temperature for the water-soluble copolymers at 1 w/w% in deionized water; model fits and deconvolutions of the SAXS data (graphs) and best fit parameters (table) from the 1 w/w% solution of P4 and P6 in deionized water at different temperatures (PDF)

## #

Hochschule Bremerhaven and guest at Alfred Wegener Institute, Am Handelshafen 12, Bremerhaven 27570, Germany

## Abbreviations

ATRP: Atom transfer radical polymerization
BuMA: *n*-butyl methacrylate
*C*_gel_: gelation concentration
CROP: cationic ring-opening polymerization
DEAEMA: 2-(diethylamino)­ethyl methacrylate
DEGMA: di­(ethylene glycol) methyl ether methacrylate
*d*_H_s: hydrodynamic diameters
DLS: dynamic light scattering
DMAEMA: 2-(dimethylamino)­ethyl methacrylate
DPPH: 2-diphenyl-1-picrylhydrazyl hydrate
EG: ethylene glycol
GTP: group transfer polymerization
LCST: lower critical solution temperature
MM: molar mass
MMD: molar mass distribution
MM^theor.^: theoretical MM
*M*_n_: number-average MM
MTS: methyltrimethylsilyl dimethylketene acetal
NMR: nuclear magnetic resonance
OEGMA300: oligo­(ethylene glycol) methyl ether methacrylate with average *M*_n_ 300 g mol^–1^
PBS: phosphate-buffered saline
PMMA: poly­(methyl methacrylate)
PTFE: poly­(tetrafluoroethylene)
RAFT: reversible addition–fragmentation chain-transfer
SEC: size exclusion chromatography
SAXS: small-angle X-ray scattering
TBABB: tetrabutylammonium bibenzoate
*T*_CP_: cloud point temperature
TEM: transmission electron microscopy
*T*_gel_: gelation temperature
THF: tetrahydrofuran
UCST: upper critical solution temperature
UV–vis: ultraviolet visible.

## References

[1] Matsukuma D., Sambai T., Otsuka H. (2017). UCST-Type Phase Transition Driven
by Protein-Derived Polypeptide Employing Gelatin and Chitosan. Polym. Adv. Technol..

[2] Constantinou A. P., Georgiou T. K. (2021). Pre-Clinical and
Clinical Applications of Thermoreversible
Hydrogels in Biomedical Engineering: A Review. Polym. Int..

[3] Pertici V., Trimaille T., Gigmes D. (2020). Inputs of Macromolecular Engineering
in the Design of Injectable Hydrogels Based on Synthetic Thermoresponsive
Polymers. Macromolecules.

[4] Zhang M., Vora A., Han W., Wojtecki R. J., Maune H., Le A. B. A., Thompson L. E., McClelland G. M., Ribet F., Engler A. C., Nelson A. (2015). Dual-Responsive
Hydrogels
for Direct-Write 3D Printing. Macromolecules.

[5] Rocha V. G., García-Tuñón E., Botas C., Markoulidis F., Feilden E., D’Elia E., Ni N., Shaffer M., Saiz E. (2017). Multimaterial 3D Printing of Graphene-Based Electrodes for Electrochemical
Energy Storage using Thermoresponsive Inks. ACS Appl. Mater. Interfaces.

[6] Constantinou A. P., Wang L., Wang S., Georgiou T. K. (2023). Thermoresponsive
Block Copolymers of Increasing Architecture Complexity: A Review on
Structure–property Relationships. Polym.
Chem..

[7] Lanzalaco S., Armelin E. (2017). Poly­(N-Isopropylacrylamide) and Copolymers: A Review
on Recent Progresses in Biomedical Applications. Gels.

[8] Vancoillie G., Frank D., Hoogenboom R. (2014). Thermoresponsive
Poly­(Oligo Ethylene
Glycol Acrylates). Prog. Polym. Sci..

[9] Concilio M., Beyer V. P., Becer C. R. (2022). Thermoresponsive Polymers in Non-Aqueous
Solutions. Polym. Chem..

[10] Sentoukas T., Pispas S. (2018). Poly­(Dimethylaminoethyl
Methacrylate)-B-Poly­(Hydroxypropyl
Methacrylate) Copolymers: Synthesis and pH/Thermo-Responsive Behavior
in Aqueous Solutions. J. Polym. Sci., Part A:
Polym. Chem..

[11] Kafetzi M., Pispas S. (2021). Multifaceted pH and Temperature Induced Self-Assembly
of P­(DMAEMA-co-LMA)-B-POEGMA Terpolymers and their Cationic Analogues
in Aqueous Media. Macromol. Chem. Phys..

[12] Shan X., Aspinall S., Kaldybekov D. B., Buang F., Williams A. C., Khutoryanskiy V. V. (2021). Synthesis
and Evaluation of Methacrylated Poly­(2-Ethyl-2-Oxazoline)
as a Mucoadhesive Polymer for Nasal Drug Delivery. ACS Appl. Polym. Mater..

[13] Hoogenboom R., Schlaad H. (2017). Thermoresponsive Poly­(2-Oxazoline)­s,
Polypeptoids,
and Polypeptides. Polym. Chem..

[14] Sugihara S., Kanaoka S., Aoshima S. (2005). Double Thermosensitive
Diblock Copolymers
of Vinyl Ethers with Pendant Oxyethylene Groups: Unique Physical Gelation. Macromolecules.

[15] Choi S. W., Choi S. Y., Jeong B., Kim S. W., Lee D. S. (1999). Thermoreversible
Gelation of Poly­(Ethylene Oxide) Biodegradable Polyester Block Copolymers.
II. J. Polym. Sci., Part A: Polym. Chem..

[16] Jung Y., Park W., Park H., Lee D., Na K. (2017). Thermo-Sensitive
Injectable Hydrogel Based on the Physical Mixing of Hyaluronic Acid
and Pluronic F-127 for Sustained NSAID Delivery. Carbohydr. Polym..

[17] Zentner G. M., Rathi R., Shih C., McRea J. C., Seo M.-., Oh H., Rhee B. G., Mestecky J., Moldoveanu Z., Morgan M. (2001). Biodegradable Block
Copolymers for Delivery of Proteins
and Water-Insoluble Drugs. J. Controlled Release.

[18] Al
Khateb K., Ozhmukhametova E. K., Mussin M. N., Seilkhanov S. K., Rakhypbekov T. K., Lau W. M., Khutoryanskiy V. V. (2016). In Situ
Gelling Systems Based on Pluronic F127/Pluronic F68 Formulations for
Ocular Drug Delivery. Int. J. Pharm..

[19] Ge Z., Zhou Y., Tong Z., Liu S. (2011). Thermogelling of Double
Hydrophilic Multiblock and Triblock Copolymers of N,N-Dimethylacrylamide
and N-Isopropylacrylamide: Chain Architectural and Hofmeister Effects. Langmuir.

[20] Chen J., Liu M., Gong H., Cui G., Lü S., Gao C., Huang F., Chen T., Zhang X., Liu Z. (2013). Synthesis
of Linear Amphiphilic Tetrablock Quaterpolymers with Dual Stimulus
Response through the Combination of ATRP and RAFT by a Click Chemistry
Site Transformation Approach. Polym. Chem..

[21] Constantinou A. P., Zhang K., SomuncuoĞlu B., Feng B., Georgiou T. K. (2021). PEG-Based
Methacrylate Tetrablock Terpolymers: How does the Architecture Control
the Gelation?. Macromolecules.

[22] Determan M. D., Cox J. P., Seifert S., Thiyagarajan P., Mallapragada S. K. (2005). Synthesis and Characterization of
Temperature and pH-Responsive
Pentablock Copolymers. Polymer.

[23] Determan M. D., Guo L., Lo C.-., Thiyagarajan P., Mallapragada S. K. (2008). pH- and
Temperature-Dependent Phase Behavior of a PEO-PPO-PEO-Based Pentablock
Copolymer in Aqueous Media. Phys. Rev. E.

[24] Popescu M.-T., Athanasoulias I., Tsitsilianis C., Hadjiantoniou N. A., Patrickios C. S. (2010). Reversible
Hydrogels from Amphiphilic Polyelectrolyte
Model Multiblock Copolymers: The Importance of Macromolecular Topology. Soft Matter.

[25] Li Z., Zhang Z., Liu K. L., Ni X., Li J. (2012). Biodegradable
Hyperbranched Amphiphilic Polyurethane Multiblock Copolymers Consisting
of Poly­(Propylene Glycol), Poly­(Ethylene Glycol), and Polycaprolactone
as in Situ Thermogels. Biomacromolecules.

[26] Sanal T., Oruç O., Öztürk T., Hazer B. (2015). Synthesis
of pH- and Thermo-Responsive Poly (E-Caprolactone-B-4-Vinyl Benzyl-G-Dimethyl
Amino Ethyl Methacrylate) Brush Type Graft Copolymers Via RAFT Polymerization. J. Polym. Res..

[27] Georgiou, T. ; Constantinou, A. W. Polymers; WO 2,020,065,295 A1, 2020.

[28] Constantinou A. P., Zhan B., Georgiou T. K. (2021). Tuning the Gelation of Thermoresponsive
Gels Based on Triblock Terpolymers. Macromolecules.

[29] Sedlacek O., Bardoula V., Vuorimaa-Laukkanen E., Gedda L., Edwards K., Radulescu A., Mun G. A., Guo Y., Zhou J., Zhang H. (2022). Influence of Chain Length of Gradient and Block Copoly­(2-Oxazoline)­s
on Self-Assembly and Drug Encapsulation. Small.

[30] Min K., Kwon Oh J., Matyjaszewski K. (2007). Preparation of Gradient Copolymers
Via ATRP in Miniemulsion. II. Forced Gradient. J. Polym. Sci., Part A: Polym. Chem..

[31] Lee S. B., Russell A. J., Matyjaszewski K. (2003). ATRP Synthesis
of Amphiphilic Random,
Gradient, and Block Copolymers of 2-(Dimethylamino)­Ethyl Methacrylate
and N-Butyl Methacrylate in Aqueous Media. Biomacromolecules.

[32] Min K., Li M., Matyjaszewski K. (2005). Preparation
of Gradient Copolymers
Via ATRP using a Simultaneous Reverse and Normal Initiation Process.
I. Spontaneous Gradient. J. Polym. Sci., Part
A: Polym. Chem..

[33] Filippov S. K., Verbraeken B., Konarev P. V., Svergun D. I., Angelov B., Vishnevetskaya N. S., Papadakis C. M., Rogers S., Radulescu A., Courtin T., Martins J. C., Starovoytova L., Hruby M., Stepanek P., Kravchenko V. S., Potemkin I. I., Hoogenboom R. (2017). Block and Gradient Copoly­(2-Oxazoline)
Micelles: Strikingly Different on the Inside. J. Phys. Chem. Lett..

[34] Sedlacek O., Lava K., Verbraeken B., Kasmi S., De Geest B. G., Hoogenboom R. (2019). Unexpected
Reactivity Switch in the Statistical Copolymerization
of 2-Oxazolines and 2-Oxazines Enabling the One-Step Synthesis of
Amphiphilic Gradient Copolymers. J. Am. Chem.
Soc..

[35] Glassner M., Lava K., de la Rosa V. R., Hoogenboom R. (2014). Tuning the
LCST of Poly­(2-Cyclopropyl-2-Oxazoline) Via Gradient Copolymerization
with 2-Ethyl-2-Oxazoline. J. Polym. Sci., Part
A: Polym. Chem..

[36] Steinhauer W., Hoogenboom R., Keul H., Moeller M. (2013). Block and Gradient
Copolymers of 2-Hydroxyethyl Acrylate and 2-Methoxyethyl Acrylate
Via RAFT: Polymerization Kinetics, Thermoresponsive Properties, and
Micellization. Macromolecules.

[37] Bera D., Sedlacek O., Jager E., Pavlova E., Vergaelen M., Hoogenboom R. (2019). Solvent-Control
Over Monomer Distribution in the Copolymerization
of 2-Oxazolines and the Effect of a Gradient Structure on Self-Assembly. Polym. Chem..

[38] Van
Steenberge P. H. M., Verbraeken B., Reyniers M., Hoogenboom R., D’hooge D. R. (2015). Model-Based Visualization and Understanding of Monomer
Sequence Formation in Gradient Copoly­(2-Oxazoline)­s on the Basis of
2-Methyl-2-Oxazoline and 2-Phenyl-2-Oxazoline. Macromolecules.

[39] Bloksma M. M., Hoeppener S., D’Haese C., Kempe K., Mansfeld U., Paulus R. M., Gohy J., Schubert U. S., Hoogenboom R. (2012). Self-Assembly
of Chiral Block and Gradient Copolymers. Soft
Matter.

[40] Hoogenboom R., Lambermont-Thijs H., Jochems M. J. H. C., Hoeppener S., Guerlain C., Fustin C., Gohy J., Schubert U. S. (2009). A Schizophrenic
Gradient Copolymer: Switching and Reversing Poly­(2-Oxazoline) Micelles
Based on UCST and Subtle Solvent Changes. Soft
Matter.

[41] Lobert M., Hoogenboom R., Fustin C., Gohy J., Schubert U. S. (2008). Amphiphilic
Gradient Copolymers Containing Fluorinated 2-Phenyl-2-Oxazolines:
Microwave-Assisted One-Pot Synthesis and Self-Assembly in Water. J. Polym. Sci., Part A: Polym. Chem..

[42] Hoogenboom R., Fijten M. W. M., Wijnans S., van den Berg A. M. J., Thijs H. M. L., Schubert U. S. (2006). High-Throughput
Synthesis and Screening
of a Library of Random and Gradient Copoly­(2-Oxazoline)­s. J. Comb. Chem..

[43] Lambermont-Thijs H., Jochems M. J. H. C., Hoogenboom R., Schubert U. S. (2009). Synthesis and Properties
of Gradient Copolymers Based on 2-Phenyl-2-Oxazoline and 2-Nonyl-2-Oxazoline. J. Polym. Sci., Part A: Polym. Chem..

[44] Bradford K. G. E., Gilbert R. D., Weerasinghe M. A. S., Harrisson S., Konkolewicz D. (2023). Spontaneous Gradients by ATRP and
RAFT: Interchangeable
Polymerization Methods?. Macromolecules.

[45] Farias-Mancilla B., Zhang J., Kulai I., Destarac M., Schubert U. S., Guerrero-Sanchez C., Harrisson S., Colombani O. (2020). Gradient and
Asymmetric Copolymers: The Role of the Copolymer Composition Profile
in the Ionization of Weak Polyelectrolytes. Polym. Chem..

[46] Zhang J., Farias-Mancilla B., Destarac M., Schubert U. S., Keddie D. J., Guerrero-Sanchez C., Harrisson S. (2018). Asymmetric Copolymers: Synthesis,
Properties, and Applications of Gradient and Other Partially Segregated
Copolymers. Macromol. Rapid Commun..

[47] Liu X., Wang M., Harrisson S., Debuigne A., Marty J., Destarac M. (2017). Enhanced Stabilization of Water/scCO_2_ Interface
by Block-Like Spontaneous Gradient Copolymers. ACS Sustainable Chem. Eng..

[48] Yañez-Macias R., Kulai I., Ulbrich J., Yildirim T., Sungur P., Hoeppener S., Guerrero-Santos R., Schubert U. S., Destarac M., Guerrero-Sanchez C., Harrisson S. (2017). Thermosensitive Spontaneous Gradient
Copolymers with Block- and Gradient-Like Features. Polym. Chem..

[49] Sykes K. J., Harrisson S., Keddie D. J. (2016). Phosphorus-Containing Gradient (Block)
Copolymers Via RAFT Polymerization and Postpolymerization Modification. Macromol. Chem. Phys..

[50] Harrisson S., Ercole F., Muir B. W. (2010). Living
Spontaneous Gradient Copolymers
of Acrylic Acid and Styrene: One-Pot Synthesis of pH-Responsive Amphiphiles. Polym. Chem..

[51] Kaberov L. I., Kaberova Z., Murmiliuk A., Trousil J., Sedláček O., Konefal R., Zhigunov A., Pavlova E., Vít M., Jirák D., Hoogenboom R., Filippov S. K. (2021). Fluorine-Containing
Block and Gradient Copoly­(2-Oxazoline)­s Based on 2­(3,3,3-Trifluoropropyl)-2-Oxazoline:
A Quest for the Optimal Self-Assembled Structure for ^19^F Imaging. Biomacromolecules.

[52] Lambermont-Thijs H. M. L., Fijten M. W. M., ton van der Linden A.
J., van Lankvelt B. M., Bloksma M. M., Schubert U. S., Hoogenboom R. (2011). Efficient
Cationic Ring-Opening Polymerization of Diverse Cyclic Imino Ethers:
Unexpected Copolymerization Behavior. Macromolecules.

[53] Webster O. W. (2000). The Discovery
and Commercialization of Group Transfer Polymerization. J. Polym. Sci., Part A: Polym. Chem..

[54] Webster, O. W. Group Transfer Polymerization: A Critical Review of Its Mechanism and Comparison with Other Methods for Controlled Polymerization of Acrylic Monomers. In New Synthetic Methods. Advances in Polymer Science; Springer: Berlin, Heidelberg, 2004; Vol. 167, pp. 1–34.

[55] Rikkou-Kalourkoti, M. ; Webster, O. W. ; Patrickios, C. S. Group Transfer Polymerization. Encycl. Polym. Sci. Technol., 2013. 10.1002/0471440264.pst603.

[56] Dicker I. B., Cohen G. M., Farnham W. B., Hertler W. R., Laganis E. D., Sogah D. Y. (1990). Oxyanions Catalyze Group-Transfer Polymerization to
Give Living Polymers. Macromolecules.

[57] Constantinou A. P., SomuncuoĞlu B., Georgiou T. K. (2024). An environmentally friendly synergistic
stabilization of emulsions using an ABC macrosurfactant and hydroxypropyl
methylcellulose. J. Polym. Sci..

[58] Blanchet C. E., Spilotros A., Schwemmer F., Graewert M. A., Kikhney A., Jeffries C. M., Franke D., Mark D., Zengerle R., Cipriani F., Fiedler S., Roessle M., Svergun D. I. (2015). Versatile
Sample Environments and Automation for Biological Solution X-Ray Scattering
Experiments at the P12 Beamline (PETRA III, DESY). J. Appl. Crystallogr..

[59] Hajizadeh N. R., Franke D., Svergun D. I. (2018). Integrated
Beamline Control and Data
Acquisition for Small-Angle X-Ray Scattering at the P12 BioSAXS Beamline
at PETRAIII Storage Ring DESY. J Synchrotron
Rad..

[60] Gruzinov A. Y., Schroer M. A., Malanastas-Cantos K., Kikhney A. G., Hajizadeh N. R., Schulz F., Franke D., Svergun D. I., Blanchet C. E. (2021). Anomalous
SAXS at P12 Beamline EMBL Hamburg: Instrumentation and Applications. J Synchrotron Rad..

[61] Petoukhov M. V., Konarev P. V., Kikhney A. G., Svergun D. I. (2007). ATSAS 2.1
–
Towards Automated and Web-Supported Small-Angle Scattering Data Analysis. J. Appl. Crystallogr..

[62] Manalastas-Cantos K., Konarev P. V., Hajizadeh N. R., Kikhney A. G., Petoukhov M. V., Molodenskiy D. S., Panjkovich A., Mertens H. D. T., Gruzinov A., Borges C. (2021). ATSAS 3.0: Expanded Functionality and New Tools for
Small-Angle Scattering Data Analysis. J. Appl.
Crystallogr..

[63] Doucet, M. ; Cho, J. H. ; Alina, G. ; Attala, Z. ; Bakker, J. ; Bouwman, W. ; Butler, P. ; Campbell, K. ; Cooper-Benun, T. ; Durniak, C. SasView version 5.0.4. 10.5281/zenodo.4467703 (Accessed 24 November 2023).

[64] Percus J. K., Yevick G. J. (1958). Analysis of Classical Statistical Mechanics by Means
of Collective Coordinates. Phys. Rev..

[65] Shibayama M., Tanaka T., Han C. C. (1992). Small Angle
Neutron Scattering Study
on Poly­(N-isopropyl acrylamide) Gels Near Their Volume-Phase Transition
Temperature. J. Chem. Phys..

[66] Pedersen J. S. (1997). Analysis
of Small-Angle Scattering Data from Colloids and Polymer Solutions:
Modeling and Least-Squares. Adv. Colloid Interface
Sci..

[67] Guinier, A. ; Fournet, G. Small-Angle Scattering of X-Rays; John Wiley and Sons: New York, 1955.

[68] Kotlarchyk M., Chen S. H. (1983). Analysis of Small Angle Neutron Scattering
Spectra
from Polydisperse Interacting Colloids. J. Chem.
Phys..

[69] Kotlarchyk M., Stephens R. B., Huang J. S. (1988). Study of Schultz Distribution to
Model Polydispersity of Microemulsion Droplets. J. Phys. Chem. A.

[70] Constantinou A. P., Nele V., Doutch J. J., Correia J. S., Moiseev R. V., Cihova M., Gaboriau D. C. A., Krell J., Khutoryanskiy V. V., Stevens M. M. (2022). Investigation of the Thermogelation of a Promising
Biocompatible ABC Triblock Terpolymer and its Comparison with Pluronic
F127. Macromolecules.

[71] Constantinou A. P., Georgiou T. K. (2016). Thermoresponsive
Gels based on ABC Triblock Copolymers:
Effect of the Length of the PEG Side Group. Polym. Chem..

[72] Ward M. A., Georgiou T. K. (2013). Multicompartment
Thermoresponsive Gels: Does the Length
of the Hydrophobic Side Group Matter?. Polym.
Chem..

[73] Li Q., Constantinou A. P., Georgiou T. K. (2021). A Library of Thermoresponsive PEG-based
Methacrylate Homopolymers: How do the Molar Mass and Number of Ethylene
Glycol Groups Affect the Cloud Point?. J. Polym.
Sci..

[74] Lutz J.-F., Akdemir Ö., Hoth A. (2006). Point by Point Comparison of Two
Thermosensitive Polymers Exhibiting a Similar LCST: Is the Age of
Poly­(NIPAM) Over?. J. Am. Chem. Soc..

[75] Okabe S., Seno K., Kanaoka S., Aoshima S., Shibayama M. (2006). Small-Angle
Neutron Scattering Study on Block and Gradient Copolymer Aqueous Solutions. Polymer.

[76] Okabe S., Seno K., Kanaoka S., Aoshima S., Shibayama M. (2006). Micellization
Study on Block and Gradient Copolymer Aqueous Solutions by DLS and
SANS. Macromolecules.

[77] Kravchenko V. S., Potemkin I. I. (2016). Micelles of Gradient
vs Diblock Copolymers: Difference
in the Internal Structure and Properties. J.
Phys. Chem. B.

[78] Coldstream J. G., Camp P. J., Phillips D. J., Dowding P. J. (2022). Gradient Copolymers *versus* Block Copolymers:
Self-Assembly in Solution and Surface
Adsorption. Soft Matter.

[79] Lutz J. (2008). Polymerization
of Oligo­(Ethylene Glycol) (Meth)­Acrylates: Toward New Generations
of Smart Biocompatible Materials. J. Polym.
Sci., Part A: Polym. Chem..

[80] Wu C., Ying A., Ren S. (2013). Fabrication
of Polymeric Micelles
with Core-Shell-Corona Structure for Applications in Controlled Drug
Release. Colloid Polym. Sci..

[81] Cui S., Yu L., Ding J. (2019). Thermogelling
of Amphiphilic Block Copolymers in Water:
ABA Type versus AB or BAB Type. Macromolecules.

[82] Ward M. A., Georgiou T. K. (2010). Thermoresponsive Terpolymers Based on Methacrylate
Monomers: Effect of Architecture and Composition. J. Polym. Sci., Part A: Polym. Chem..

[83] Constantinou A. P., Sam-Soon N., Carroll D. R., Georgiou T. K. (2018). Thermoresponsive
Tetrablock Terpolymers: Effect of Architecture and Composition on
Gelling Behavior. Macromolecules.

