# Supplementary material for: Thermogels Based on Block versus Gradient Terpolymers: Differences in the Nano- and Macro-Scale
Source: Macromolecules. 2025 Aug 26;58(17):9122–39. doi: 10.1021/acs.macromol.5c00821 (PMC12424292; doi:10.1021/acs.macromol.5c00821)
Supplement: Supplementary file 1 [file ma5c00821_si_001.pdf]

Supporting Information

Thermogels based on block *versus* gradient terpolymers: Differences in the nano- and macro-scale

Anna P. Constantinou,[a] Feifei Zheng,[b] Lezhi Wang,[a] Wenqi Xu,[b] Qian Li,[a] Beini Zhan,[a] Joana Salvado Correia,[a] Birsen Somuncuoğlu,[a] Stefano Da Vela,[c] Christine M. Papadakis,[b] Theoni K. Georgiou[a,*]*

[a] Department of Materials, Imperial College London, London, SW7 2AZ, United Kingdom (UK).

[b] Technical University of Munich, TUM School of Natural Sciences, Physics Department, Soft Matter Physics Group, James-Franck-Str. 1, 85748 Garching, Germany.

[c] European Molecular Biology Laboratory, Hamburg Site, c/o Deutsches Elektronen-Synchrotron, Hamburg 22607, Germany; Present Address: Hochschule Bremerhaven and guest at Alfred Wegener Institute, Am Handelshafen 12, Bremerhaven 27570, Germany.

*Prof Theoni K. Georgiou: t.georgiou@imperial.ac.uk

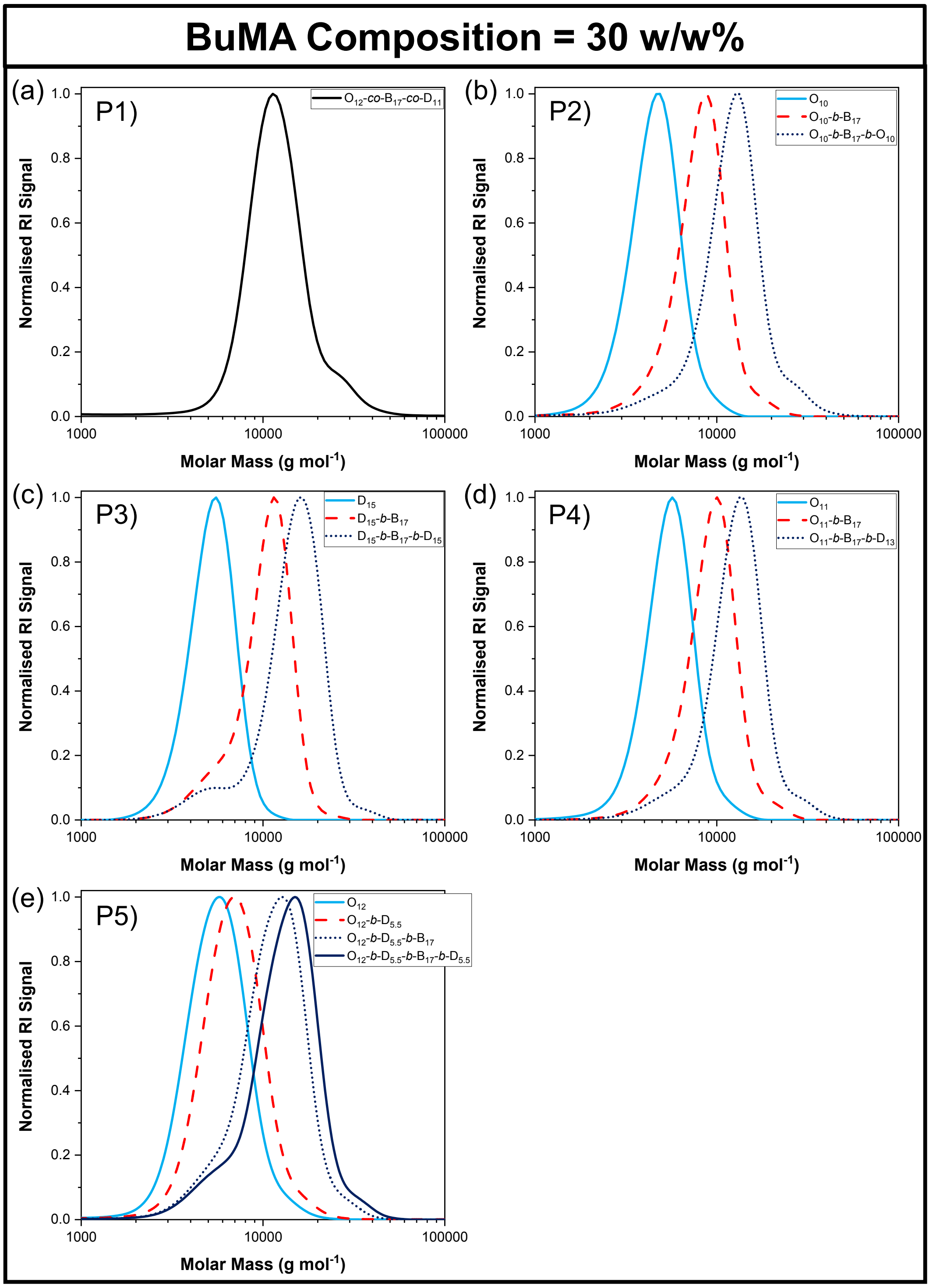

Figure S1. SEC chromatograms of the copolymers with 30 w/w% BuMA. Regarding the SEC traces of the block copolymers, the first, second, third, and fourth (if any) blocks are shown in

light blue solid, red dashed, dark blue dotted, and dark blue solid lines, respectively, while the one of the statistical terpolymer (Polymer 1) is shown in black solid line.

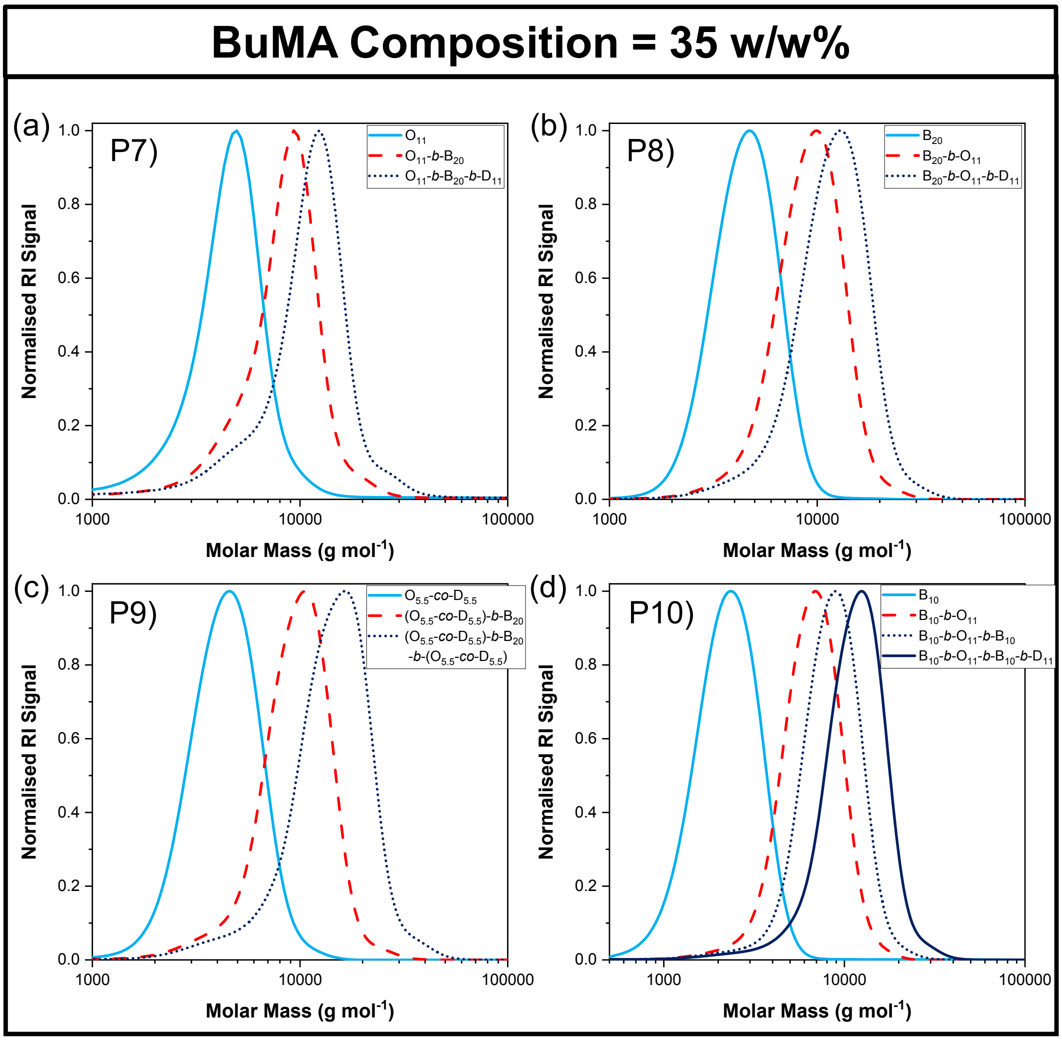

Figure S2. SEC chromatograms of the copolymers with 35 w/w% BuMA. Regarding the SEC traces of the block copolymers, the first, second, third, and fourth (if any) blocks are shown in light blue solid, red dashed, dark blue dotted, and dark blue solid lines, respectively.

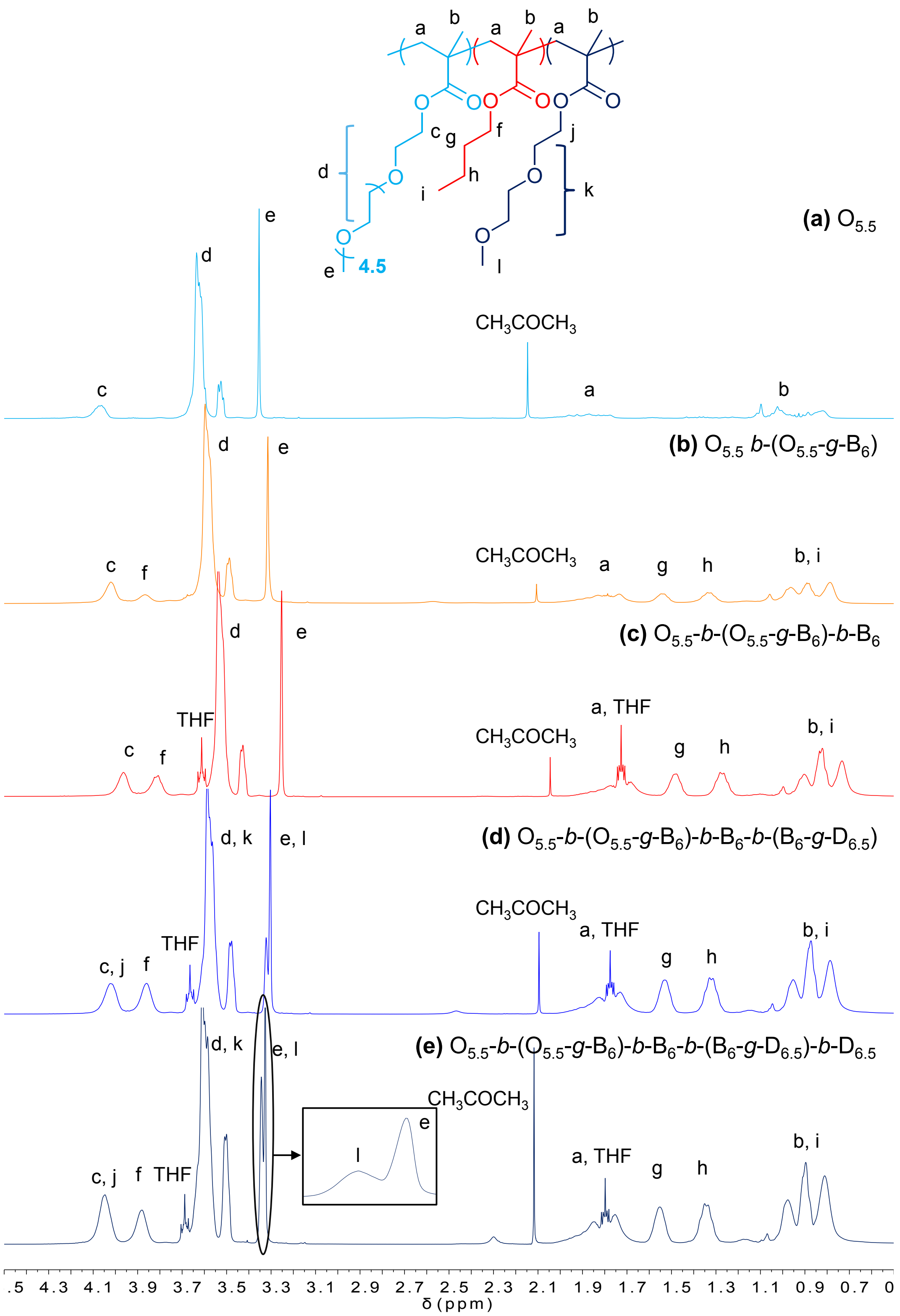

Figure S3. ^{1}H NMR spectra of the gradient terpolymer, OEGMA300$_{5.5}$-*b*-(OEGMA300$_{5.5}$-*g*-BuMA$_{6}$)-*b*-BuMA$_{6}$-*b*-(BuMA$_{6}$-*g*-DEGMA$_{6.5}$)-*b*-DEGMA$_{6.5}$ (e, Sample 11) in dark blue and its precursors: (a) OEGMA300$_{5.5}$ in light blue (Sample 1), (b) OEGMA300$_{5.5}$-*b*-(OEGMA300$_{5.5}$-*g*-BuMA$_{6}$) in orange (Sample 5), OEGMA300$_{5.5}$-*b*-(OEGMA300$_{5.5}$-*g*-BuMA$_{6}$)-*b*-BuMA$_{6}$ in red

(Sample 6), and OEGMA300$_{5.5}$-*b*-(OEGMA300$_{5.5}$-*g*-BuMA$_6$)-*b*-BuMA$_6$-*b*-(BuMA$_6$-*g*-DEGMA$_{6.5}$) in blue (Sample 10). The general chemical structure is also shown, with full annotation of the protons of different chemical environment in both the chemical structure and the ^{1}H NMR spectra.

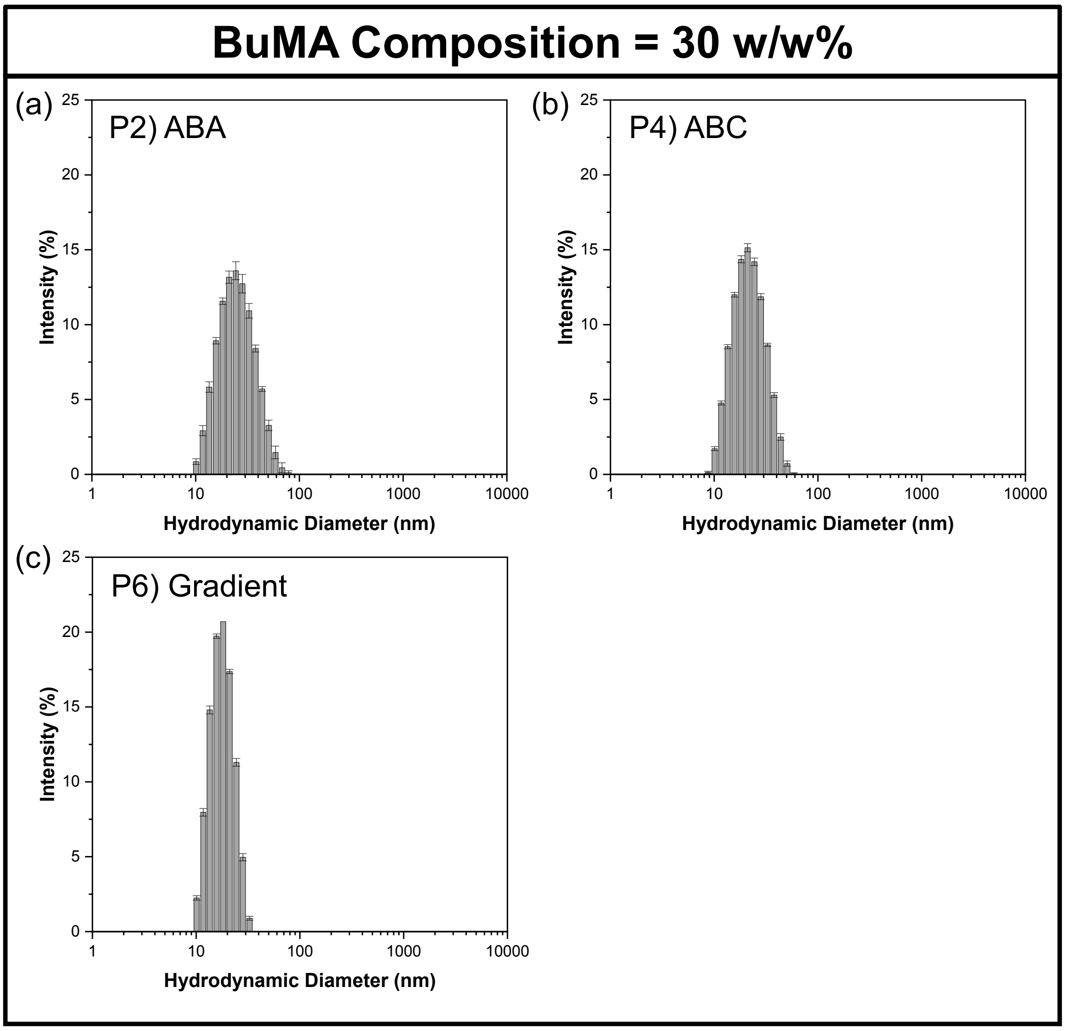

Figure S4. DLS histograms by intensity of the aqueous solutions of the copolymers with 30 w/w% hydrophobic BuMA content at 1 w/w% in deionized water and 25 °C.

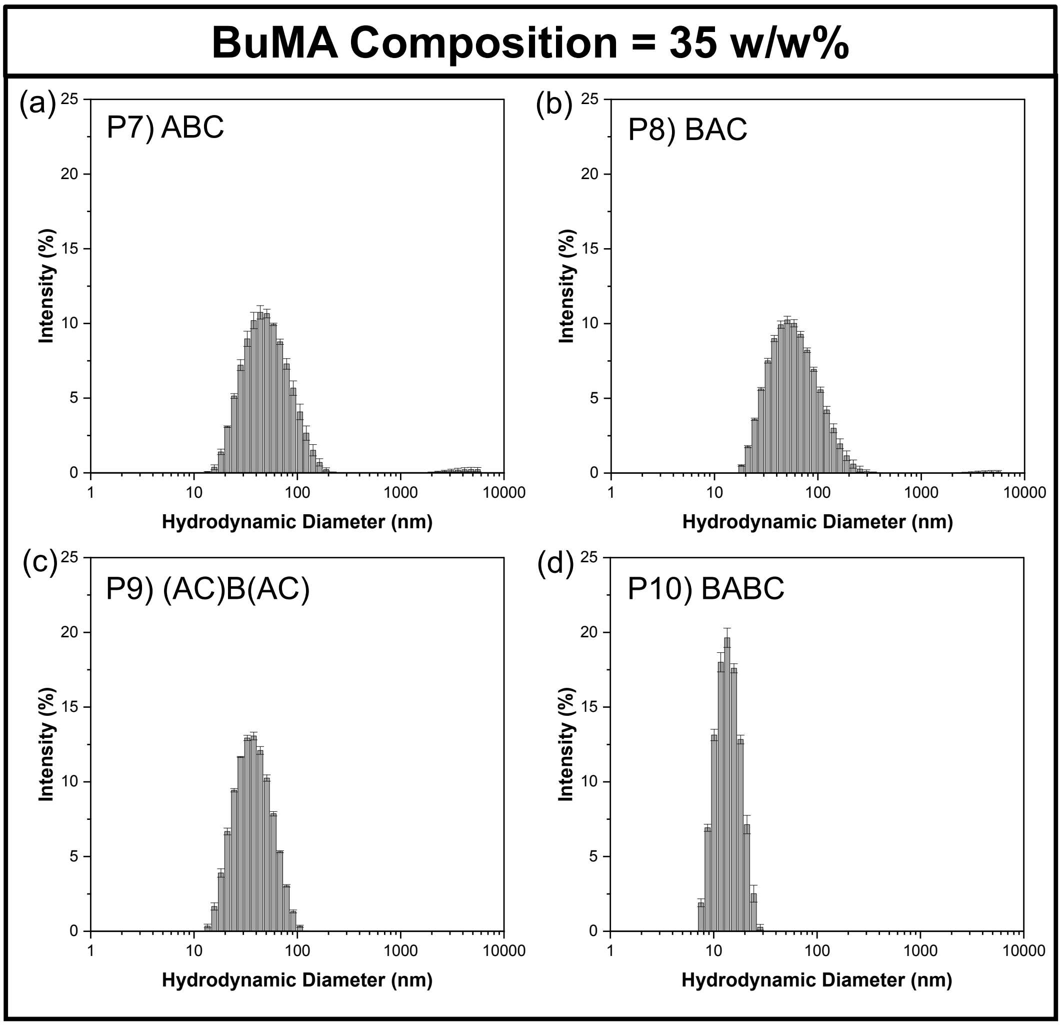

Figure S5. DLS histograms by intensity of the aqueous solutions of the copolymers with 35 w/w% hydrophobic BuMA content at 1 w/w% in deionized water and 25 ˚C.

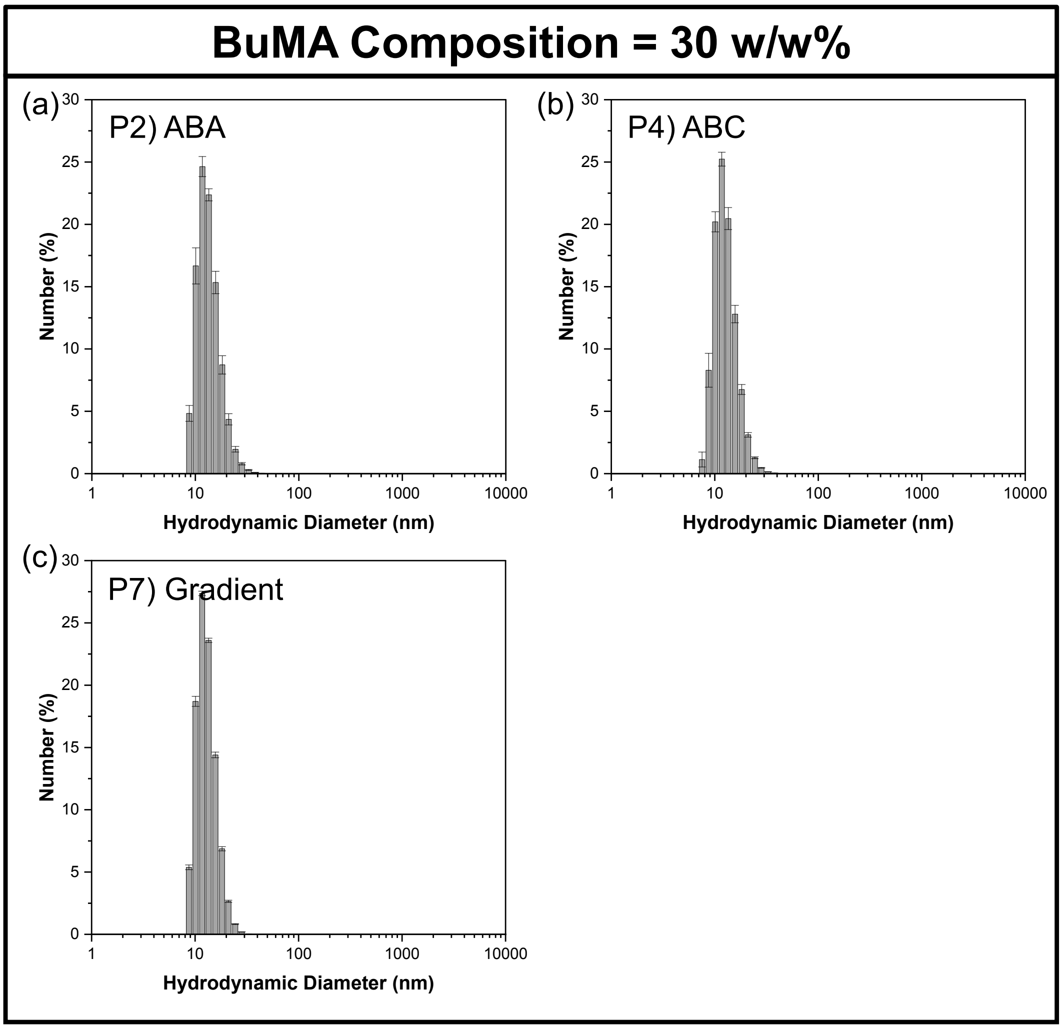

Figure S6. DLS histograms by number of the aqueous solutions of the copolymers with 30 w/w% hydrophobic BuMA content at 1 w/w% in deionized water and 25 °C.

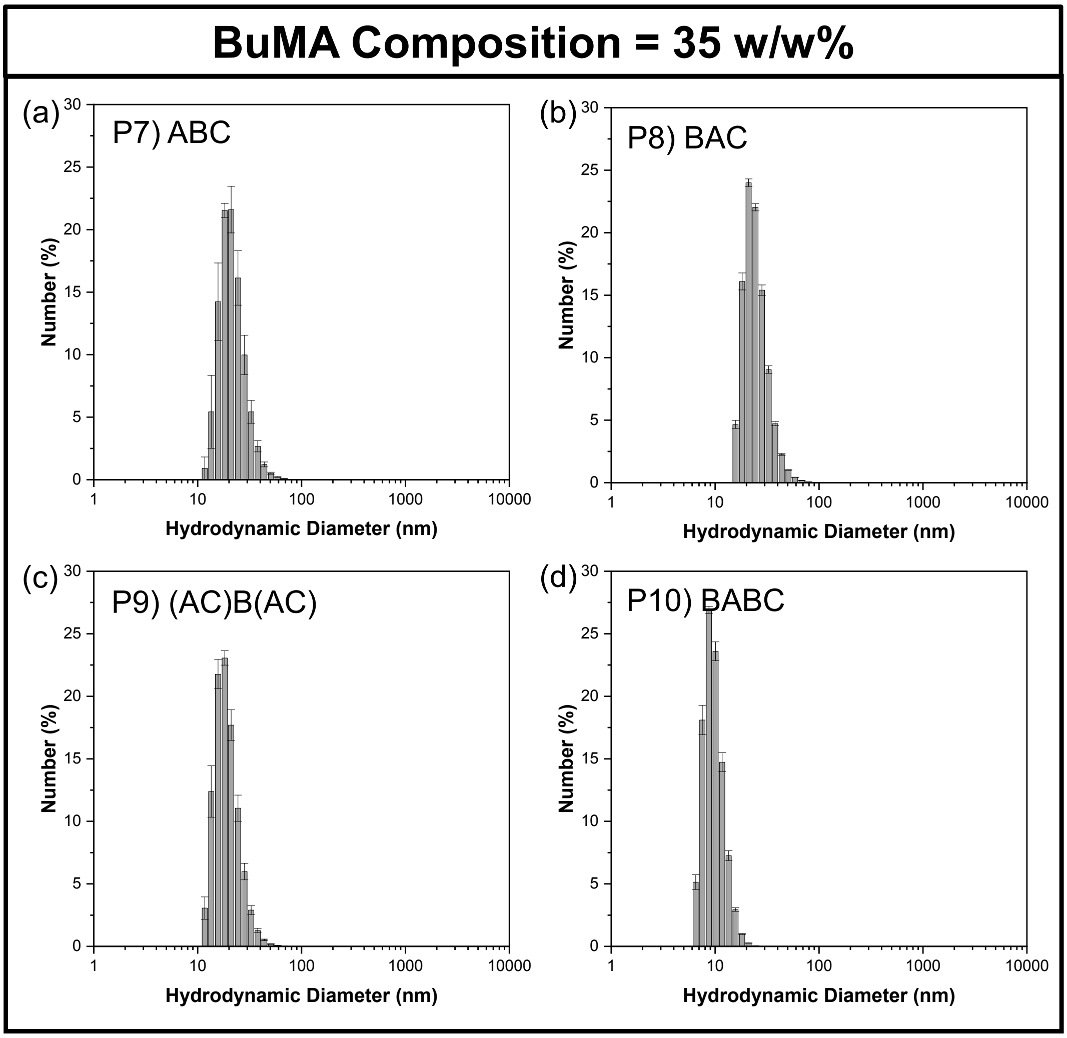

Figure S7. DLS histograms by number of the aqueous solutions of the copolymers with 35 w/w% hydrophobic BuMA content at 1 w/w% in deionized water and 25 °C.

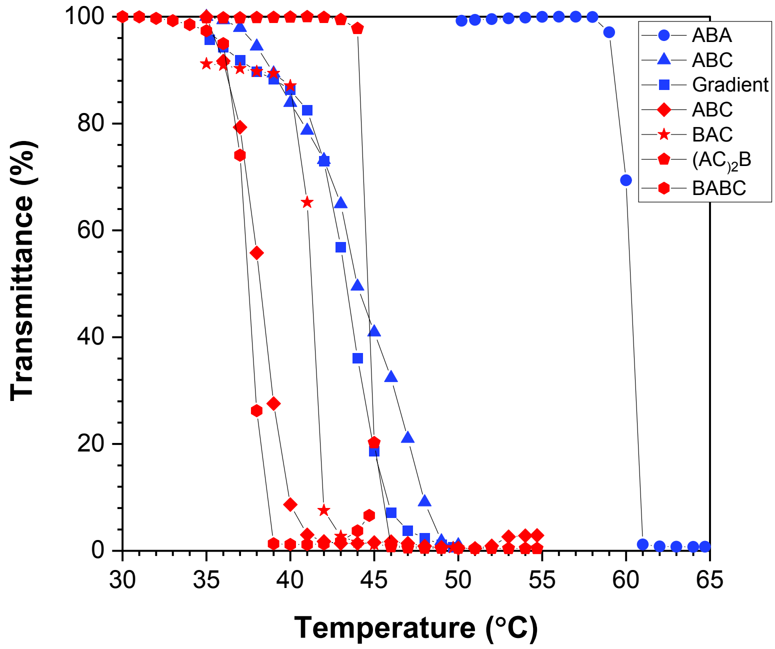

Figure S8. Transmittance as a function of temperature for the water-soluble copolymers at 1 w/w% in deionized water. The results that correspond to the ABA, ABC, and gradient copolymers, with 30 w/w% BuMA, are indicated by blue circles, triangles, and squares, respectively. The results of the ABC, BAC, $(AC)_2B$, and BABC copolymers, with 35 w/w% BuMA, are shown in red diamonds, stars, pentagons, and hexagons, respectively.

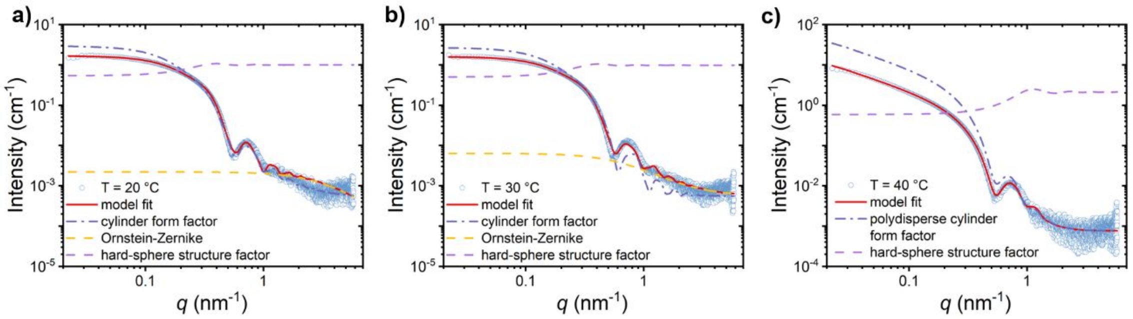

Figure S9. Model fits and deconvolutions of the SAXS data from the 1 w/w% solution of the triblock terpolymer ABC (Polymer 4) in deionized water at (a) 20 ˚C, (b) 30 ˚C and (c) 40 ˚C. Blue circles: data points, red full lines: overall model fits, dashed lines: contributions from the single terms in the fitting model, as detailed in the legends.

Table S1. Best fit parameters of Equation 1 for the SAXS data of the 1 w/w% solution of the triblock terpolymer ABC (Polymer 4) in deionized water at different temperatures.

Temperature (˚C)	20	30	40
R_{cyl} [nm]	6.94 ± 0.01	6.95 ± 0.40	7.08 ± 0.01
Polydispersity σ_{cyl}	0	0	0.01± 0.00
L_{cyl} [nm]	33.8 ± 0.05	33.7 ± 0.04	364 ± 4
η	0.09 ± 0.01	0.09 ± 0.01	0.02 ± 0.01
R_{HS} [nm]	7.33 ± 0.01	7.10 ± 0.02	2.66 ± 0.02
ξ_{OZ} [nm]	1.29 ± 0.03	1.45 ± 0.04	- [a)]
ρ_{cyl} [10^{-6} Å^{-2}]	9.60 ± 0.00	9.60 ± 0.00	9.62 ± 0.01
χ^2 [b)]	3.6	5.7	1.8

a) The Ornstein-Zernike term is not needed.

b) The least-squares method employs the chi-squared function as a measure for data quality.

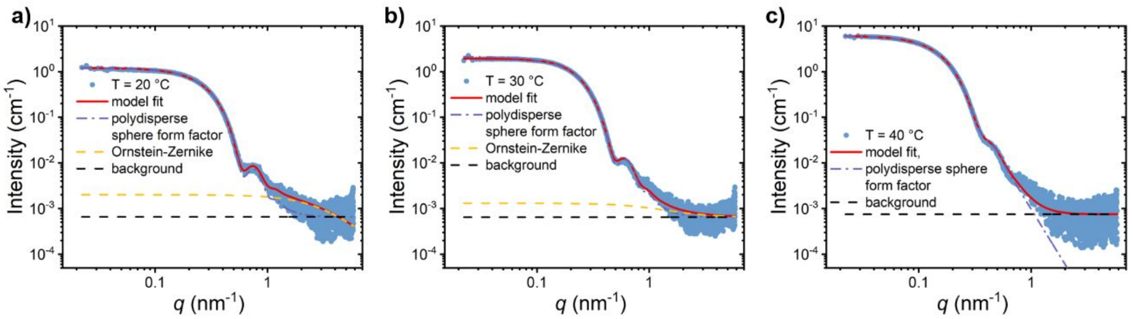

Figure S10. Model fits of the SAXS data from the 1 w/w% solution of the gradient terpolymer (Polymer 6) in H_2O at (a) 20˚C, (b) 30˚C and (c) 40 ˚C. Blue circles: data points, red full lines: overall model fits, dashed lines: contributions from the single terms in the fitting model, as detailed in the legends.

Table S2. Best fit parameters of Equation 1 for the SAXS data of the 1 w/w% solution of the gradient terpolymer (Polymer 6) in deionized water at different temperatures.

Temperature (˚C)	20	30	40
R_m [nm]	7.26 ± 0.01	8.83 ± 0.00	11.56 ± 0.01
Polydispersity σ_{sph}	0.11 ± 0.00	0.12 ± 0.00	0.20 ± 0.01
ξ_{OZ} [nm]	1.28 ± 0.01	0.85 ± 0.01	- [a)]
ρ_{sph} [10^{-6} Å^{-2}]	9.60 ± 0.00	9.61 ± 0.00	9.61 ± 0.01
χ^2 [b)]	2.1	4.0	1.8

a) The Ornstein-Zernike term is not needed.

b) The least-squares method employs the chi-squared function as a measure for data quality.
